# The maturation of native uropathogenic *Escherichia coli* biofilms seen through a non-interventional lens

**DOI:** 10.1016/j.bioflm.2024.100212

**Published:** 2024-07-06

**Authors:** Tianqi Zhang, Sanhita Ray, Keira Melican, Agneta Richter-Dahlfors

**Affiliations:** a AIMES-Center for the Advancement of Integrated Medical and Engineering Sciences, Karolinska Institutet and KTH Royal Institute of Technology, SE-171 77, Stockholm, Sweden; b Department of Neuroscience, Karolinska Institutet, SE-171 77, Stockholm, Sweden

**Keywords:** Biofilm, UPEC, Curli, Optotracing, EbbaBiolight, 2-Photon, Wettability

## Abstract

Urinary tract infections (UTI) caused by uropathogenic *Escherichia coli* (UPEC) are a significant global health challenge. The UPEC biofilm lifestyle is believed to play an important role in infection recurrency and treatment resistance, but our understanding of how the extracellular matrix (ECM) components curli and cellulose contribute to biofilm formation and pathogenicity is limited. Here, we study the spatial and temporal development of native UPEC biofilm using agar-based detection methods where the non-toxic, optically active fluorescent tracer EbbaBiolight 680 reports the expression and structural location of curli in real-time. An *in vitro* screen of the biofilm capacity of common UPEC strains reveals significant strain variability and identifies UPEC No. 12 (UPEC12) as a strong biofilm former at 28 °C and 37 °C. Non-interventional microscopy, including time-lapse and 2-photon, reveal significant horizontal and vertical heterogeneity in the UPEC12 biofilm structure. We identify region-specific expression of curli, with a shift in localization from the bottom of the flat central regions of the biofilm to the upper surface in the topographically dramatic intermediate region. When investigating if the rdar morphotype affects wettability of the biofilm surface, we found that the nano-architecture of curli guided by cellulose, rather than the rdar macrostructures, leads to increased hydrophobicity of the biofilm. By providing new insights at exceptional temporal and spatial resolution, we demonstrate how non-interventional analysis of native biofilms will facilitate the next generation of understanding into the roles of ECM components during growth of UPEC biofilms and their contribution to the pathogenesis of UTI.

## 1 Introduction

Urinary tract infection (UTI) will affect between 50 and 60 % of adult women during their lifetime and around 25 % will experience a recurrence within 6 months. The most common causative bacterial agent is uropathogenic *Escherichia coli* (UPEC). The formation of biofilm by UPEC is believed to contribute to recurrency by allowing bacterial persistence in the urinary tract. An extracellular biofilm-like lifestyle has been shown to be important for bacterial colonization in the lumen of the proximal tubule of the kidney [1], whereas an intracellular life-style has been suggested within the bladder epithelium [2]. In patients with catheter-associated urinary tract infections (CAUTIs) biofilm formation leads to significant treatment complications [3]. The formation of biofilm is a considerable clinical issue, in terms of increased antibiotic resistance and treatment failure but also due to the negative economic impact [4].

Each bacterial species develops a unique biofilm whose components and structures vary dependent on the microenvironment in which they form [5]. A common theme, however, is the extracellular matrix (ECM) which provides structural support to the biofilm and protection to the bacterial cells. For *E. coli* and its close relative *Salmonella*, the ECMs are dominated by the fibril-forming amyloid protein curli and the polysaccharide cellulose [[6], [7], [8]]. Analysis of mature biofilm phenotypes can be subjectively defined according to morphotypes on agar containing dyes such as congo red and crystal violet, known as rdar (red, dry, and rough). These morphotypes have shown that curli and cellulose form characteristic ridges in the biofilm [6]. It is still unclear, however, how the spatial and temporal expression of curli and cellulose and the interactions between them, lead to ridge formation, as well as what function these structures fulfill. Understanding how, when, and why ECM components are expressed during biofilm formation would enhance the possibilities of targeting these components in the next generation of biofilm disrupting treatments.

The need for methods allowing dynamic, quantitative, real-time studies of native biofilm formation spurred us to develop the optotracing technique using *Salmonella* Enteritidis as model system [8]. Optotracing is based on a group of oligothiophene-based fluorescent molecules whose photophysical properties change upon binding to amyloid proteins and polysaccharides [9,10]. With a unique ability to label the native structures of such macromolecules, we applied optotracing for direct detection of the presence of biofilm in urine from UTI patients, highlighting the importance of the biofilm lifestyle during clinical UPEC infection, and the potential for optotracing as a clinical diagnostic tool for biofilm infections [11]. Building on the next generation optotracers, the EbbaBiolight series, we quantified and monitored in real-time growth of bacterial cells and ECM production during *Salmonella* Enteritidis biofilm formation on EbbaBiolight 680-containing agar (Ebba680-agar) [10]. We identified the kinetics of biofilm formation on agar, showing distinct regions in the macrocolony identified as the inner core, the intermediate region, and the outer region. We identified curli production as essential to the development of distinct radial structures of the intermediate region [10]. An innovative use of Ebba680-agar also formed the basis of the first antibiofilm-specific antibiotic susceptibility test (AST), in which the effects of antibiotics on bacterial growth and ECM production within biofilms were visualized and quantified [12]. Biofilm formation by other microbes, such as *Burkholderia cenocepacia*, *Klebsiella pneumoniae, Pseudomonas aeruginosa* and *Candida albicans* has also been addressed using optotracing approaches [10,[12], [13], [14], [15], [16], [17], [18]].

Bacterial species show vastly different biofilm forming ability, and this variability even extends to strains within a species. CFT073, a widely used strain in UTI and biofilm research, was used by our lab in intravital studies of UPEC kidney infections, identifying an important role of biofilm for successful establishment of infection *in vivo* [19,20]. But CFT073 shows poor rdar morphotypes on CR-containing agar (CR-agar), suggesting low biofilm-forming capacity *in vitro* [21]. Our recently established link between respiratory nitrate reduction and biofilm formation showed, however, that the biofilm-forming capacity of CFT073 is greatly improved when grown on nitrate-supplemented agar [21]. Other UPEC strains, such as UPEC No.12 (UPEC12), isolated from a child with pyelonephritis, do however show strong biofilm formation on CR-agar [22]. This illustrates the great variability in inherent biofilm-forming capacity amongst UPEC strains, which varies further depending on the *in vitro* growth conditions used. This demonstrates that a wide variety of biofilm model systems are needed to better understand the complexity of the biofilm lifestyle.

In this work, we use non-interventional techniques to analyze the spatial and temporal development of native biofilms forming at 37 °C. From a collection of UPEC strains, we focus on UPEC12 to study biofilm heterogeneity associated with curli fibril expression. Using live automated microscopy, 2-photon microscopy, and molecular analysis, we profile the regio-specific kinetics of architectural elements in 2D and 3D. We link this architecture to the genetic expression of ECM compounds, and we examine a role for the ECM nano-architecture, synergistically formed by cells, curli fibrils and cellulose, in providing a water excluding surface for the biofilm.

## 2 Materials and methods

### 2.1 Bacterial strains, media, and supplements

Bacterial strains and plasmids used are listed in Supplementary Table 1. To enable stable GFP expression, wild type (wt) and isogenic mutants of UPEC No.12 (UPEC12) were transformed with the GFP-reporter plasmid pFPV25.1 [23], generating strains UPEC12-pFPV25.1 (UPEC12-GFP), UPEC12 *ΔbcsA*-pFPV25.1 (UPEC12 *ΔbcsA*-GFP) and UPEC12 *ΔcsgBA*-pFPV25.1 (UPEC12 *ΔcsgBA*-GFP). UPEC12 is part of the sequence type ST12 [24] and the draft genome of this strain can be found at GenBank: MIIG00000000.1ac [25]. Strains were cultured in Luria-Bertani (LB) broth without (w/o) salt (10 g/l tryptone, 5 g/l yeast extract (Sigma-Aldrich, Stockholm, Sweden)) or on LB agar w/o salt (15 g/l of Bacto™ Agar (BD Biosciences, Franklin Lakes, New Jersey, USA) added to the LB broth w/o salt) at 37 °C. For biofilm assays, 10 μl of overnight cultures grown in LB broth w/o salt (37 °C, 180 rpm) were drop inoculated onto LB agar w/o salt, supplemented with EbbaBiolight 680 (Ebba680, 0.5 μl/ml) (Ebba Biotech, Stockholm, Sweden), or Congo red (CR, 40 μg/ml, Sigma-Aldrich, Stockholm, Sweden) together with Coomassie brilliant blue G-250 (20 μg/ml; Sigma-Aldrich, Stockholm, Sweden). When using strains containing the GFP reporter plasmid, Ampicillin (Amp, 100 μg/ml) was added. Plates were incubated at 28 °C or 37 °C as indicated.

### 2.2 Congo red biofilm assay

The Congo red biofilm assay (CR-biofilm assay) was performed in accordance with Choong et al. [10] In short, 2 ml CR-containing agar (CR-agar), prepared by supplementing LB agar w/o salt with CR and Coomassie brilliant blue G-250, was added per well in sterile, Costar® tissue culture treated 6-well plates (Sigma-Aldrich, Stockholm, Sweden). 10 μl overnight bacterial culture was inoculated onto the center of each well and allowed to dry. Plates were incubated at 28 °C or 37 °C for 72 h. Morphotypes based on curli and cellulose expression were determined by visual inspection and documented by photography.

### 2.3 Ebba680-based biofilm assay

In the biofilm assay based on Ebba680 (Ebba680-biofilm assay), 2 ml LB agar w/o salt supplemented with the tracer molecule Ebba680 (Ebba680-agar) was added per well to sterile, Costar® tissue culture treated 6-well plates (Sigma-Aldrich, Stockholm, Sweden). 100 μg/ml Amp was added when required. Biofilm formation was initiated by placing 10 μl overnight culture onto the center of each well. Once the inoculum had dried, plates were incubated at 28 °C or 37 °C for 72 h, either in an incubator or in the automated microscope (Lionheart™ FX Automated Microscope (Ramcon, Stockholm, Sweden).

### 2.4 Automated microscopy for live imaging of biofilms on Ebba680 agar

Fluorescence imaging of biofilms (cells and ECM) in the Ebba680-biofilm assay was performed with a Lionheart™ FX Automated Microscope and the Gen5 software (Ramcon, Stockholm, Sweden). For real-time monitoring of biofilm growth, the Lionheart™ FX Automated Microscope was programmed to allow automatic imaging for up to 72 h at 1 h intervals in a constant 28 °C or 37 °C environment. To image the entire biofilm, a 1.25x objective, Plan Apochromat WD 5 NA 0.04 was used. Fluorescence from Ebba680 was imaged using a 523 nm LED cube/propidium iodide (PI) filter cube, GFP fluorescence was detected using a 465 nm LED cube/GFP filter cube, and the contour of the macrocolonies were recorded by brightfield. The LED intensity, integration time and gain were 10; 132; 20 for Ebba680 detection, 5; 100; 0 for GFP detection, and 5; 100; 0 for brightfield image recording. Images were collected as a 6-by-5 grid that was stitched together during post imaging analysis. The brightfield image was used as a reference signal for auto-focusing and stitching. Colony measurements were performed using Gen5 software based on the brightfield images. For statistical analysis, two-way ANOVA with Tukey's multiple comparison test was used in GraphPad Prism 10 (Graph-pad Software, La Jolla, CA, USA)

### 2.5 Quantification of ECM in biofilms formed on Ebba680 agar

Quantification of fluorescence intensities of Ebba680 within biofilms was performed by placing the Ebba680-biofilm assay plate in an Infinite® M1000 Tecan microplate spectrophotometer (Tecan, Männedorf, Switzerland). To acquire the total relative fluorescence units (RFU) of the entire biofilm, fluorescence was measured at Ex. *λ* 535 nm, Em. *λ* 660 nm with 9 nm bandwidth using a circle multiple read of 15 x 15. Statistical difference was determined with one-way ANOVA with Tukey's multiple comparison test using GraphPad Prism 10 (Graph-pad Software, La Jolla, CA, USA).

### 2.6 RNA isolation, cDNA synthesis, and qPCR

Following 18 h incubation at 37 °C, entire biofilms were scraped from LB agar w/o salt using inoculation loops (VWR, Stockholm, Sweden). Alternatively, samples from the central core and the intermediate region were isolated separately. Isolated biomass were resuspended in 1 ml nuclease-free water (not DEPC-treated) in RNase-free microfuge tubes (Thermo Fisher Scientific, Sweden). After a brief vortex, biofilms were pelleted by centrifugation (4000 rpm, 5 min, 4 °C). RNA was isolated using the PureLink™ RNA Mini Kit (Thermo Fisher Scientific, Stockholm, Sweden) following manufacturer's protocol. Purity and yield of the total RNA was confirmed before qPCR. PureLink™ DNase Set (Thermo Fisher Scientific, Stockholm, Sweden) removed DNA contamination. The concentration and purity of RNA isolates were analyzed with a NanoDrop™ 1000 Spectrophotometer (Thermo Fisher Scientific, Stockholm, Sweden) and integrity confirmed by gel electrophoresis.

cDNA synthesis was performed with SuperScript™ III First-Strand Synthesis SuperMix (Thermo Fisher Scientific, Stockholm, Sweden). PCR analysis was performed using RT2 SYBR® Green qPCR Mastermixes (Qiagen, Stockholm, Sweden) with QuantStudio™ 5 (Thermo Fisher Scientific, Stockholm, Sweden) following the manufacturer's protocol with minor modifications. Results were analyzed and presented with GraphPad Prism 10 (Graph-pad Software, La Jolla, CA, USA). For comparison of expression levels, comparative CT analysis (ΔΔCT method) was employed with *rpoD* as housekeeping gene. Expressions were normalized against specific data sets. For comparison between different *E. coli* strains, expressions were normalized against UPEC12 and statistical differences were analyzed by one-way ANOVA using GraphPad Prism 10 (Graph-pad Software, La Jolla, CA, USA). For the comparison between regions in UPEC12 biofilm, expressions were normalized against the central region and statistical differences were analyzed with unpaired *t*-test using GraphPad Prism 10 (Graph-pad Software, La Jolla, CA, USA).

### 2.7 Multiphoton fluorescence imaging of cells and curli in biofilms on Ebba680-agar

Multiphoton imaging of the native biofilm was achieved by transferring the UPEC12-GFP biofilm, which had grown on the Ebba680-biofilm assay at 37 °C for 72 h, onto an ibidi μ-Dish 35 mm, high (ibidi, Gräfelfing, Germany). Without further treatment (no fixation, no additional staining, no added coverslip), a quarter of the biofilm was imaged using a dry objective (HC PL APO 10x/0.40 CS2 10x) on a Leica SP8 DIVE multiphoton microscope (Leica Microsystems, Wetzlar, Germany). To detect fluorescence from Ebba680 bound to curli, we used 800 nm excitation laser at 2 % intensity, and a PMT detector with emission filter 650 nm–710 nm, digital gain of 800 v. For GFP detection, an excitation laser of 930 nm at 7 % intensity was used, and a HyD detector with emission filter 480 nm–550 nm, digital gain of 100 %. The image data was processed and presented with Leica Application Suite X (Leica Microsystems, Wetzlar, Germany). Imaris 10.0 (Oxford Instruments, Abingdon, United Kingdom) was used to crop and render the acquired multiphoton fluorescence images. Surface function was used to present the detailed architecture of the structures of the biofilm.

### 2.8 Wettability test

The wettability characteristics of the central and intermediate regions of native biofilms, formed by UPEC12-GFP, UPEC12 *ΔbcsA*-GFP, and UPEC12 *ΔcsgBA*-GFP on Ebba680-agar in 6-well plates during 72 h at 37 °C, were analyzed by placing the biofilm and its supportive agar onto a smooth black plastic surface. A PGX pocket goniometer (Fibro system, Stockholm, Sweden) was placed on top of the biofilm, with care taken to ensure exact positioning of the droplet needle. By pressing the fill button, the instrument dropped approximately 2 μl of MilliQ water onto the biofilm surface. Using the built-in software, the contact angle and the final volume of the droplet were calculated based on the droplet shape (height and base width). On the rare occasion that images contained high background, the droplet base and height were adjusted manually for accuracy. Statistical differences from 3 biological repeats were analyzed with one-way ANOVA using GraphPad Prism 10 (Graph-pad Software, La Jolla, CA, USA).

### 2.9 Statistical analysis

Statistical comparisons were performed and presented with GraphPad Prism 10 (Graph-pad Software, La Jolla, CA, USA). Details of the statistical methods used for each specific data set is described in the corresponding sections.

### 2.10 Preparation and analysis of images

Fiji software (v1.52 h) (Wisconsin, USA) [26] was used to process and perform orthogonal analysis of image stacks acquired by confocal microscopy, and to analyze the physical dimensions of biofilms in images collected by automated microscopy. When analysing the kinetics of region-specific biofilm formation in Fig. 4, the fluorescence channel images of each 72 h image series were sorted separately and analyzed with MATLAB R2020a (Version: 9.8.0.1873465) and image processing toolbox (Version 11.1). Using the 72 h red fluorescence image as reference, four regions of interest (ROIs) (10-by-10 pixels each) were chosen within the central, center-intermediate, intermediate and outer regions. The average green and red pixel intensities in each ROIs were calculated for all the images over 72 h. MATLAB codes for calculating kinetics and for annotating the 72 h image with ROIs are available on GitHub (https://github.com/AIMES-ARD/UPECPaper) Images for final presentation were prepared by Adobe Illustrator (Adobe, San Jose, USA).

## 3 Results

### 3.1 Optotracing reveals strain diversity of biofilm formation

**Fig. 1:**
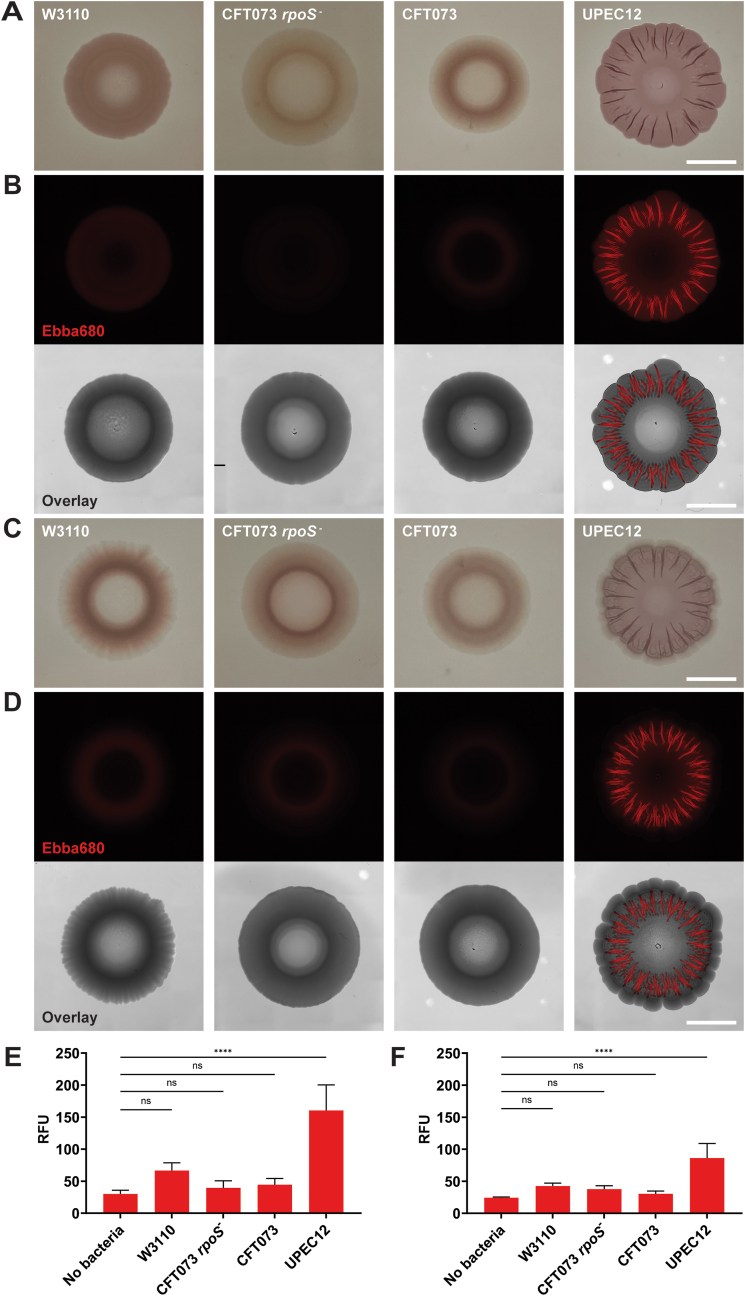
Biofilm morphotypes on Congo red- and Ebba680-biofilm assays. Biofilms formed by annotated strains grown for 72 h. (A) Photographs of *E. coli* on CR-agar at 28 °C. (B) Wide-field fluorescence imaging of *E. coli* on Ebba680-biofilm assay at 28 °C. The Ebba680 (red) image, obtained in the propidium iodide channel, is shown separately and in overlay with the brightfield image. Scale bar = 5 mm. Representative of n = 3. (C) Photographs of *E. coli* on CR-agar at 37 °C. (D) Wide-field fluorescence imaging of *E. coli* on Ebba680-biofilm assay at 37 °C. The Ebba680 (red) image, obtained in the propidium iodide channel, is shown separately and in overlay with the brightfield image. Scale bar = 5 mm. Representative of n = 3. (E, F) Quantitative total RFU measurement of *E. coli* on Ebba680-biofilm assay at (E) 28 °C or (F) 37 °C. Fluorescence of Ebba680 was measured at Ex. 530 nm, Em. 650 nm. Mean with SD is shown. n = 3. Statistical significance was determined by one-way ANOVA. Statistical significance is denoted by ns (not significant) or ****p ≤ 0.0001. (For interpretation of the references to colour in this figure legend, the reader is referred to the Web version of this article.)

As optotracing offers a new fluorescence-based technique for biofilm research, we started by comparing biofilm morphotypes of selected UPEC strains using optotracing and Congo red assays. Strains included CFT073 *rpoS*^*-*^, CFT073, UPEC12, as well as the K-12 strain W3110. In a CR-agar biofilm assay, performed at 28 °C for 72 h, visual inspection identified UPEC12 as the only strain demonstrating the distinct rdar (red, dry, and rough) morphology (Fig. 1A). We then performed a biofilm assay using the non-toxic fluorescent tracer molecule EbbaBiolight 680 (Ebba680) as an agar supplement [27]. Colony morphologies in the Ebba680-biofilm assay were analyzed using fluorescence wide-field microscopy (Ex. *λ* 531/40 nm, Em. *λ* 647/57 nm). UPEC12 displayed distinct radial structures highlighted by fluorescence from ECM-bound Ebba680 while other strains gave almost no discernible signal (Fig. 1B). This pattern was even more pronounced when the fluorescent signal was overlayed with bright field illumination, showing the location of the fluorescent radial structures within the UPEC12 macrocolony. The two biofilm assays showed great complementarity, both identified UPEC12 as the most competent biofilm former of the tested strains.

To correlate this finding to physiologically relevant temperature, we analyzed the biofilm-forming capability of strains at 37 °C. Each strain showed morphotypes similar to those observed at 28 °C for both the CR- and Ebba680-biofilm assays (Fig. 1C and D). With strong rdar morphotype and Ebba680 fluorescence, this identified UPEC12 as a strain with strong capacity to form biofilm at 37 °C. At 37 °C, UPEC12 formed an additional peripheral layer without fluorescent radial structures, in contrast to 28 °C.

Ebba680 lacks inherent fluorescence, the molecule only becomes fluorescent when bound to a target [8,10]. This enabled us to quantify the biofilm forming capacity of each strain by spectrophotometric recording of the total RFU of Ebba680 in each macrocolony. After 72 h incubation at 28 °C or 37 °C, the Ebba680-biofilm assay was read using a microplate reader, with RFU from each macrocolony recorded with Ex. *λ* 530 nm and Em. *λ* 650 nm. UPEC12 biofilms showed increased RFU at 28 °C as well as 37 °C, while no differences were observed between strains W3110, CFT073 *rpoS*^*-*^, and CFT073 compared to the negative control (Fig. 1E and F). This verified the ECM-producing ability of UPEC12 at both temperatures, albeit at a lower magnitude at 37 °C. UPEC12 was unique amongst the chosen strains, displaying distinct macrocolony structures at both temperatures. To align our work with the clinical relevance of UPEC biofilm *in vivo*, we focused our subsequent experiments on the physiologically relevant temperature of 37 °C.

### 3.2 The fluorescent phenotype of UPEC12 biofilm depends on curli and cellulose

**Fig. 2:**
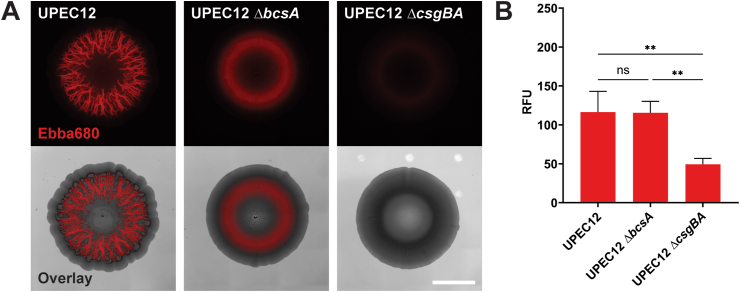
Biofilms formed by UPEC12 and isogenic mutants in the Ebba680-biofilm assay (A) Wide-field fluorescence imaging of UPEC12, UPEC12 *ΔbcsA* (curli+, cellulose-) and UPEC12 *ΔcsgBA* (curli-, cellulose+) on Ebba680-biofilm assay at 37 °C for 72 h. The Ebba680 image, representing Ebba680-labeled curli (red) imaged in the propidium iodide channel, is shown separately and in overlay with the brightfield image. Scale bar = 5 mm. Representative of n = 3. (B) Quantitative total RFU measurement of UPEC12 and isogenic mutants. Ebba680 fluorescence was measured at Ex. 530 nm, Em. 650 nm using a circle multiple read. Mean with SD shown. n = 3. Statistical significance determined by one-way ANOVA. Statistical significance denoted by ns (not significant) or **p ≤ 0.01. (For interpretation of the references to colour in this figure legend, the reader is referred to the Web version of this article.)

To identify the ECM components generating the fluorescent phenotype of UPEC12 biofilms on Ebba680-agar, we tested isogenic mutants lacking expression of the ECM components cellulose (UPEC12 *ΔbcsA)* or curli (UPEC12 *ΔcsgBA*), whose morphotypes on CR and calcofluor white agar are shown in Supplementary Fig. 1. In the Ebba680-biofilm assay (37 °C, 72 h), UPEC12 *ΔbcsA* (curli +, cellulose -) displayed substantial red fluorescence in the intermediate region of the biofilm, however, the distinct radial structures were not observed (Fig. 2A). The curli mutant UPEC12 *ΔcsgBA* (curli -, cellulose +) showed negligible fluorescence across the biofilm. Spectrophotometric quantification showed equivalent RFU from UPEC12 and UPEC12 *ΔbcsA* (curli +, cellulose -), despite the lack of radial structures in the mutant (Fig. 2B). In contrast, the curli mutant UPEC12 *ΔcsgBA* (curli -, cellulose +), showed substantially lower fluorescence intensity. Collectively, this defines curli as the primary target of Ebba680 in the Ebba680-biofilm assay. Also, it indicates that similar levels of curli are produced by UPEC12 wt and UPEC12 *ΔbcsA* (curli +, cellulose -), demonstrating an essential role for coordinated curli and cellulose expression to form the radial structures of the UPEC12 biofilm.

### 3.3 Temporal resolution of the development of morphologically distinct regions

**Fig. 3:**
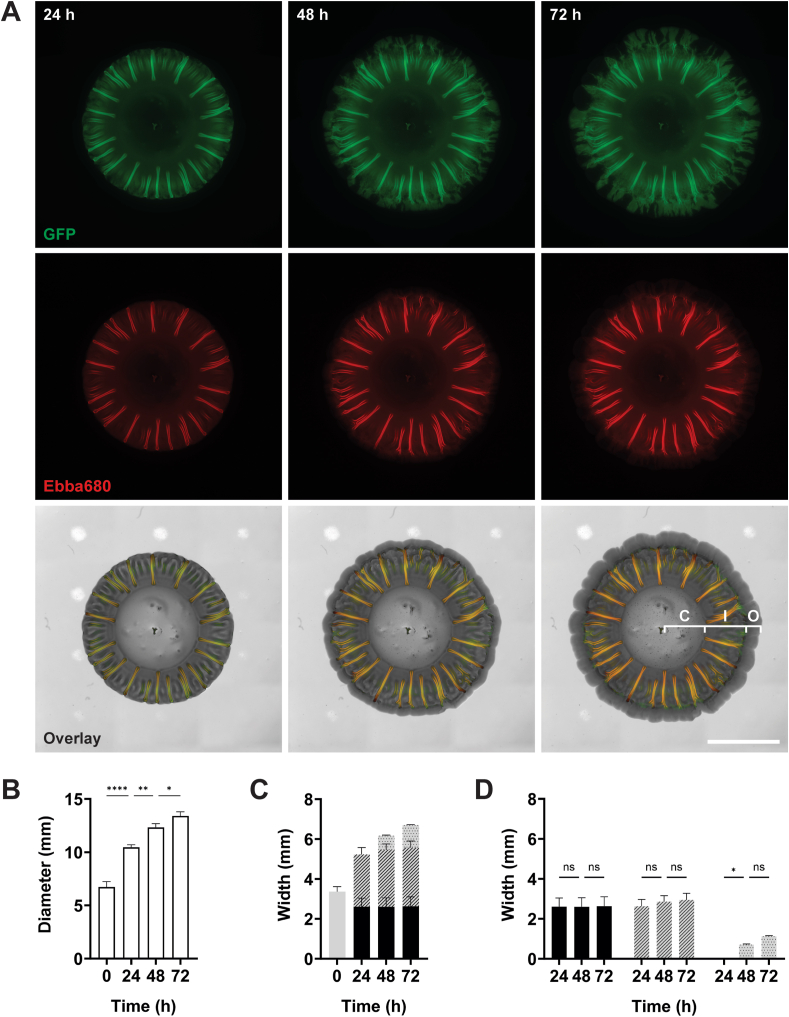
Temporal kinetics of the UPEC12 biofilm formation. Sequential imaging of UPEC12-GFP in the Ebba680-biofilm assay, showing images of the biofilm formed after 24, 48 and 72 h at 37 °C. (A) Wide-field fluorescence imaging of an individual UPEC12-GFP biofilm showing the spatial distribution of UPEC12-GFP (green, GFP channel) and Ebba680-labeled curli (red, propidium iodide channel) separately and in overlays with respective brightfield image. The widths of the central (C), intermediate (I), and outer (O) regions of end-stage biofilms are indicated in the 72 h overlay image. Scale bar = 5 mm. Representative image of n = 3. (B–C) Measurements of (B) the total biofilm diameter (n = 3, one-way ANOVA), and (C) the radius of the initial inoculum (grey, 0 h), and the width of the central (black), intermediate (striped) and outer (dotted) regions at 24, 48, and 72 h n = 3 (D) Statistical analysis (two-way ANOVA) of (C), showing the different regions separately, at indicated timepoints. Statistical significance denoted by ns (not significant), *p ≤ 0.05, **p ≤ 0.01 or ****p ≤ 0.0001. (For interpretation of the references to colour in this figure legend, the reader is referred to the Web version of this article.)

To better understand how the radial structures are formed during the development of UPEC12 biofilms, we used real-time microscopy of biofilm formation on Ebba680-agar as recently described [10]. As Ebba680 emits in the red to far-red spectra, imaging is compatible with GFP. Furthermore as fluorescence from Ebba680 is binding-induced, the signal remains off until a binding target appears. In combination, this enables real-time fluorescence recordings of the growth of GFP-expressing bacterial cells on Ebba680-agar, with simultaneous detection of curli in the growing biofilm. To enable fluorescent co-detection, we introduced a GFP-expressing plasmid to UPEC12 (UPEC12-GFP). Biofilm formation was initiated by placing 10 μl of UPEC12-GFP onto the Ebba680-biofilm assay, which was incubated in the automated microscope at 37 °C. Brightfield and fluorescence images collected at 0, 24, 48, and 72 h revealed an outward expansion of the biofilm (GFP fluorescence) and production of curli fibrils (Ebba680 fluorescence) (Fig. 3A). Three distinct morphological regions of the biofilm could be identified, named here the central, intermediate, and outer regions (annotated on the 72 h overlay of Fig. 3A). The GFP signal was heterogenous in the different regions of the biofilm, most likely due to the automatic thresholding of the microscope. In the outer region, brightfield imaging revealed a grey ring structure circling the UPEC biofilm. This is likely a photophysical effect caused by scattering of light transmitted through the agar, however, a biological phenomenon related to bacterial growth cannot be excluded. The biofilm diameter increased by 57 % over the first 24 h (from 6.7 mm to 10.5 mm) (Fig. 3B). From 24 h, the biofilm continued to grow, reaching 13.4 mm at 72 h. The flat central region showed less fluorescence than the intermediate region, which was enriched with distinct, curli-containing radial structures highlighted by Ebba680 (Fig. 3A). Biofilms imaged at 24 h, 48 h, and 72 h showed similar patterns, although the outer region was only apparent in the post 24 h biofilms. This outer region showed green fluorescence, indicating the presence of metabolically active, GFP-producing bacteria without expression of ECM.

We then analyzed the development of the morphologically distinct central, intermediate, and outer regions. From a radius of 3.29 mm of the initial inoculum, the radius of the central region was reduced to 2.61 mm at 24 h, a size that remained throughout the full length of the experiment (72 h) (Fig. 3C). The intermediate region primarily formed during the first 24 h, reaching a width of 2.62 mm. No major expansion of this region occurred after this timepoint. By 48 h, the outer region had emerged with a mean width of 0.71 mm. This region continued to expand, reaching 1.13 mm width at the 72 h endpoint. Statistical analysis demonstrated that the central and intermediate regions had reached their maximum size by 24 h (Fig. 3D). In contrast, the outer region grew significantly between 24 h and 48 h and continued to do so until 72 h. Taken together, this identified the first 24 h as an active growth phase, with establishment of the flat central region and the highly structured intermediate region. A smaller radius of the central region at 24 h compared to the inoculum suggests that formation of the curli-containing radial structures in the intermediate region are formed bi-directionally. From 24 h, growth in the horizontal plane occurred primarily in the outer region.

### 3.4 Spatial-temporal kinetics of biofilm development in the horizontal plane

**Fig. 4:**
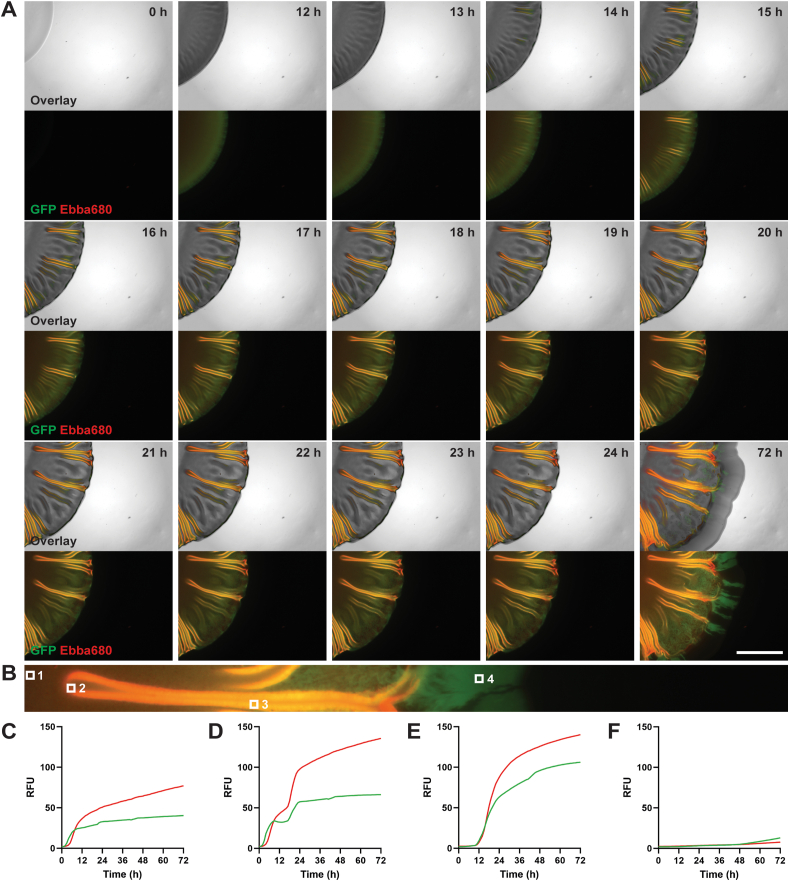
Spatial-temporal kinetics of UPEC12 biofilm development in the horizontal plane. Time-lapse wide-field fluorescence imaging of UPEC12-GFP biofilm on Ebba680-biofilm assay grown for 72 h at 37 °C. (A) Sequential images, originating from Supplementary video 1, of a part of an individual macrocolony at specified time points. The spatial distribution of UPEC12-GFP (green, GFP channel) and Ebba680-labeled curli (red, propidium iodide channel) separately and in overlays with respective brightfield image. Scale bar = 2 mm. (B) Fluorescent image of the uppermost radial structures in the 72 h biofilm in (A) selected for kinetic analysis. Squares show the positions of four (1–4) region of interests (ROI) selected from the fully formed biofilm. (C–F) Fluorescence kinetics of the ROIs with average pixel intensity (RFU) shown for (C) ROI 1, (D) ROI 2, (E) ROI 3 and (F) ROI 4. Two complementary repeats are shown in Supplementary Figs. 4 and 5 with corresponding Supplementary videos 2 and 3. (For interpretation of the references to colour in this figure legend, the reader is referred to the Web version of this article.)

Having identified the time interval when the radially structured intermediate region develops, we next focused on the spatial-temporal relationship between bacterial growth phases and ECM formation using real-time imaging. We incubated UPEC12-GFP on the Ebba680-biofilm assay at 37 °C inside an automated microscope and collected brightfield and fluorescence images hourly for 72 h. A clear outward expansion of the biofilm was observed by visualizing bacteria by GFP-expression and curli fibrils by Ebba680 fluorescence (Fig. 4A, Supplementary Video 1). While bacterial growth expanded the area covered on Ebba680-agar (exemplified at 12 h versus 0 h in Fig. 4A), red curli fluorescence in the emerging radial structures of the intermediate region only started to appear around 14 h. Radial structure formation originated from the center of the intermediate region and extended bi-directionally: outwards, in the direction of growth, as well as inwards, towards the central region. Co-localization of green and red fluorescence showed the intermediate region to be densely populated with bacteria engaged in extensive formation of curli-containing radial structures. At approximately 24 h, the intermediate region appeared to be fully formed. By 48 h, the outer region had become apparent (Supplementary Fig. 2) and continued to expand until the 72 h endpoint (Fig. 4A). The outer region appeared predominantly green, suggesting limited curli expression in this region.

Supplementary video related to this article can be found at https://doi.org/10.1016/j.bioflm.2024.100212

The following is/are the supplementary data related to this article.

Multimedia component 2 Multimedia component 2

To quantify the spatial-temporal relationship between bacterial growth and region-specific ECM formation, we performed image analyses. Based on the mature biofilm at 72 h, we defined regions of interests (ROI) representing the central region (ROI 1), the boundary between the central and intermediate region (ROI 2), the intermediate region (ROI 3), and the outer region (ROI 4) (Fig. 4B). The respective location of ROI 1–4 on the originally inoculated agar at 0 h is shown in Supplementary Fig. 3. In each 10-by-10 pixel ROI, we analyzed the average green (bacterial cells) and red (curli) pixel intensity in images collected hourly during the 72 h biofilm formation, thereby providing the kinetics of the appearance of bacteria and curli in the specific regions of the biofilm (Fig. 4C–F, upper panels). Analysis of data from one recording is shown in Fig. 4, with two repeats (Supplementary Figs. 4 and 5) and corresponding videos (Supplementary Videos 2 and 3) shown as Supplementary information.

Supplementary video related to this article can be found at https://doi.org/10.1016/j.bioflm.2024.100212

The following is/are the supplementary data related to this article:

Multimedia component 3 Multimedia component 3

Multimedia component 4 Multimedia component 4

In ROI 1, the central region, the average green and red fluorescence intensity kinetics both showed initial logarithmic increases (Fig. 4C). Green fluorescence started to increase prior to red, showing that bacterial growth preceded curli expression. For ROI 2, located at the boundary between the central and intermediate region, a steep increase in green fluorescence was initially observed, which preceded the red fluorescence by a few hours (Fig. 4D). This kinetics mimicked that observed in ROI 1, thus supporting an initial behavior of ROI 2 as part of the central region. After 7 h, the green fluorescence plateaued. At 16 h, a second phase was initiated showing a strong rise in both green and red fluorescence. These increases may result from initiation of local bacterial multiplication and associated curli expression. Alternatively, biophysical forces may be involved, such as the previously described buckling [28], which would rapidly bring large amounts of biomass into the area. In experimental repeats, the same bi-phasic kinetics were observed (Supplementary Figs. 4 and 5). Collectively, this suggests multiple processes at various stages of biofilm formation. The initial outwards growth of bacteria from the central droplet is later followed by bi-directional expansion of the radial structures likely due to a combination of bacterial multiplication, curli expression, and biophysical forces. The formation of the complex intermediate region occurs rapidly, within the 14–24 h time frame in this model. In ROI 3, in the intermediate region, cells and curli were not present until approximately 10 h–12 h (Fig. 4E). Once bacteria colonized this location, logarithmic increases of green and red fluorescence intensities were observed. The average RFUs for cell numbers and ECM content showed typical saturation kinetics with a plateau reached around 36 h. Bacterial colonization of ROI 4, in the outer region, started at approximately 48 h. An increase in green fluorescence intensity, showing the initial phase of exponential growth, occurred from 48 h to 72 h. The average RFUs were, however, much lower compared to the intermediate zone, and a fractal outer region primarily consisting of bacterial cells was formed.

By monitoring the horizontal kinetics of biofilm formation on agar in real-time, we identified the dynamics and heterogeneity of growth in the different biofilm regions. These unique insights were reliant on the non-interventional, real-time Ebba680-biofilm assay, as kinetic information is lacking from traditional end-point assays.

### 3.5 Coordinated gene expression is required for region-specific radial structure formation

**Fig. 5:**
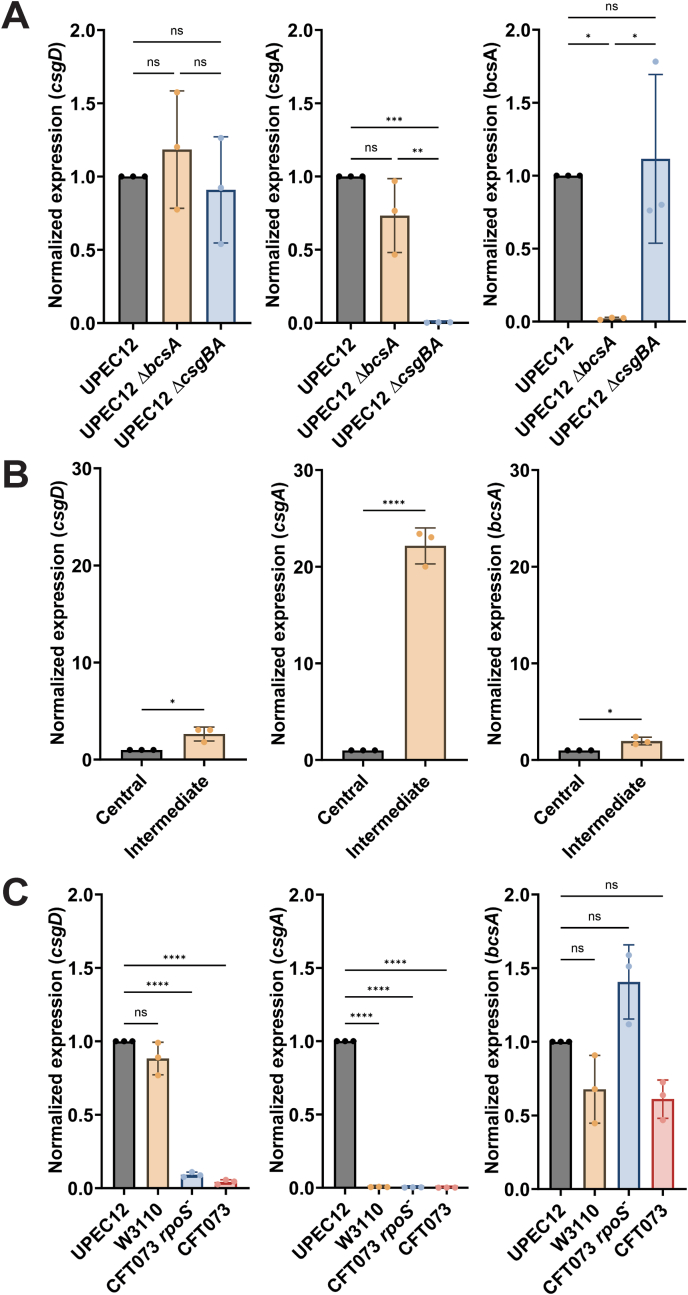
Gene expression of ECM components in *E. coli* biofilms. qPCR comparative CT analysis of *csgD*, *csgA* and *bscA* gene expression in *E. coli* biofilm at 18 h at 37 °C. (A) Analysis of biofilm macrocolonies from UPEC12 and isogenic mutants UPEC12 *ΔbcsA* and UPEC12 *ΔcsgBA*. Expression normalized against UPEC12. (B) Regio-specific analysis of UPEC12 biofilms showing gene expression in the central and intermediate regions separately. Expression normalized against central region. (C) Analysis of gene expression of annotated *E. coli* strains. Expression normalized against UPEC12. Statistical significance determined by one-way ANOVA (multiple comparisons, A, C) or *t*-test (direct comparisons, B). Dots represent individual data points, statistical significance denoted by ns (not significant), *p ≤ 0.05, **p ≤ 0.01, ***p ≤ 0.001 or ****p ≤ 0.0001. n = 3.

To better understand the role of bacterial gene expression in the formation of the phenotypically distinct regions, we investigated the expression of genes involved in ECM formation. Having identified 14 h–24 h as the primary time span for radial structure formation in the intermediate region, we harvested biofilms formed by UPEC12, UPEC12 *ΔbcsA* (curli+, cellulose-) and UPEC12 *ΔcsgBA* (curli-, cellulose+) after 18 h incubation at 37 °C. Based on total RNA, we performed qPCR analysis for genes encoding the biofilm master regulator *csgD* [29], *csgA* which controls expression of the primary curli subunit [30], and *bcsA* which controls expression of the catalytic subunit of cellulose synthase [30]. No differences were found in *csgD* expression between any of the strains (Fig. 5A). For *csgA*, no significant difference was observed between UPEC12 and UPEC12 *ΔbcsA* (curli+, cellulose-), while UPEC12 *ΔcsgBA* (curli-, cellulose+) showed the expected lack of expression. For *bcsA,* a major difference was found between UPEC12 and UPEC12 *ΔbcsA* (curli+, cellulose-), with the latter showing minimal expression. In contrast, no significant difference was observed between UPEC12 and UPEC12 *ΔcsgBA* (curli-, cellulose+). In addition to validating the genotypes and phenotypes of mutant strains, this analysis verified expression of curli and cellulose in UPEC12 biofilm at 37 °C.

We next investigated differential curli and cellulose expression in the flat central region and the highly structured intermediate region. After 18 h growth at 37 °C, we harvested biomass specifically from the two regions of UPEC12 biofilms, isolated the RNA, and performed qPCR for *csgD*, *csgA,* and *bcsA*. Significantly higher expression of all genes was observed in the intermediate region when normalized to the central region expression (Fig. 5B). The highest percentage increase was seen for *csgA*, reaching 20 times higher expression in the intermediate versus central regions. This supports our microscopy observation of extensive curli expression in the intermediate region, compared to the central region. Combined with our imaging analysis, the molecular data demonstrates a direct link between curli expression, Ebba680 fluorescence, and radial structure formation.

Intrigued by the distinct biofilm behavior of UPEC12, we analyzed our initial panel of strains (W3110, CFT073 *rpoS* ^*-*^*,* and CFT073), which in the Ebba680-biofilm assay had shown no or weak red fluorescence (Fig. 1). From each strain, we harvested the entire macrocolonies after 18 h at 37 °C, prepared RNA, and conducted qPCR analysis with expression levels normalized against UPEC12. CFT073 *rpoS*^*-*^ and CFT073 showed significantly lower levels of the biofilm master regulator *csgD* compared to UPEC12, while W3110 showed similar levels (Fig. 5C). All strains showed dramatically lowered expression of *csgA* compared to UPEC12, while no significant differences were observed in *bcsA* expression. The molecular data again supports our microscopy-based results from the Ebba680-biofilm assay, showing the biofilm phenotype of UPEC12 can be associated to high curli production. This molecular data also demonstrates the genetic heterogeneity between UPEC ECM expression at 37 °C.

### 3.6 Non-interventional workflows enable 3D structural analysis of native UPEC12 biofilms by intravital imaging

While our analysis of the spatial-temporal development of UPEC12 biofilms in the horizontal plane had given information on the topography of different regions of the biofilm, the 3D architecture remained elusive. Addressing this is challenging since the biofilms’ native structure must be retained throughout the full analytical procedure. To achieve this, we established a non-interventional workflow with the following features: i) Easy transfer of native biofilms onto a microscope slide. By growing biofilms on agar in the 6-well plate format, the entire agar can be lifted out of the well and placed on an imaging dish without compromising the biofilm; ii) No need for external staining or fixation procedures. By fluorescence, bacterial cells are identified by GFP expression, while curli is fluorescently labeled *in situ* by the agar-supplement Ebba680; iii) The photo-physics of GFP and Ebba680 are compatible with any fluorescence imaging systems, allowing us to analyze the biofilm by 2-photon microscopy; iv) Avoidance of coverslips, which may compromise the biofilm structures, is achievable in 2-photon microscopy in which dry objective with long working distance is used; v) Large-area imaging and 3D reconstruction at high resolution is achieved by 2-photon microscopy in which stitching is used to combine multiple stacks.

**Fig. 6:**
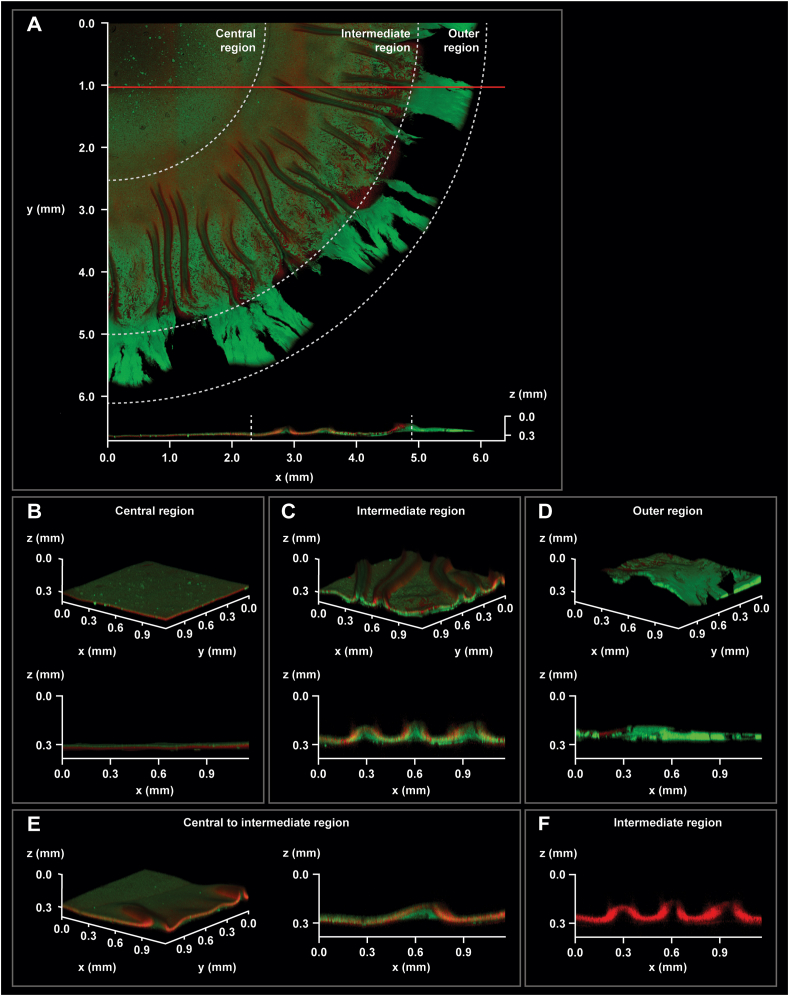
Non-interventional intravital imaging of native UPEC12-GFP biofilm. Intravital 2-photon microscopy of native UPEC12-GFP biofilm grown at 37 °C. The spatial distribution of bacterial cells (green) and Ebba680-labeled curli (red) are shown in 3D reconstructions from obtained Z-stacks. (A) 3D montage showing the xy plane of a section of the biofilm with the morphologically distinct central, intermediate, and outer regions indicated (top). Red line shows the position from which an axial cross-section, showing the xz plane of the biofilm, was generated (bottom). Vertical dotted lines indicate the borders between the central/intermediate and intermediate/outer regions. (B–E) 3D reconstruction of the xyz plane (top) and xz plane image (bottom) of the (B) central, (C) intermediate, (D) outer and (E) the bridging central to intermediate regions. (F) Axial cross-section (xz) of the red channel only, showing the curli topography in the intermediate region. Scale bars shown on individual images. All panels show representative images from n = 3. (For interpretation of the references to colour in this figure legend, the reader is referred to the Web version of this article.)

To study the details of the biofilm architecture, we grew UPEC12-GFP biofilm on Ebba680-agar in 6-well plates. After 72 h at 37 °C, we transferred the agar onto an imaging dish, which was mounted in a 2-photon microscope. We collected 6 x 6 optical stacks, covering a quarter of the biofilm and generated a 3D overview image in which the morphologically distinct central, intermediate, and outer regions were readily identifiable (Fig. 6A). Bacterial GFP expression was seen in all regions, whereas the radial structures, highlighted by red fluorescence from Ebba680, were formed primarily in the intermediate region. Using this microscopy approach, the GFP expression was uniform in the central region. The outer region showed heterogenous localization of bacterial cells, with no discernible red fluorescence.

To evaluate the topography of the native biofilm, we generated an axial cross-section (xz plane) at the position indicated by the red line in Fig. 6A. The axial cross-section (Fig. 6A bottom) revealed a flat central region, in which curli, identified by red fluorescence, was primarily found at the bottom of the biofilm, i.e. towards the agar. At the transition to the intermediate region of the biofilm, the axial cross-section demonstrated a substantial thickening of the biofilm with curli (red fluorescence) now primarily found at the air interface. The outer region showed the emergence of a GFP-expressing bacterial layer lacking red fluorescence.

We next analyzed the detailed 3D structure of each individual region of the native biofilm by constructing 3D projections from the central, intermediate, and outer regions. The central region showed a homogeneous, flat topography with evenly distributed green and red fluorescence (Fig. 6B). The cross-section of this region again revealed stratification of the biofilm with curli predominantly located beneath the surface-oriented bacterial GFP signal. A distinctly different topography was observed in the intermediate region, where the increased thickness of the radial structures dominated the landscape (Fig. 6C). The shifted location of curli was again highlighted in the cross-section of the intermediate region, with red fluorescence translocating from the base of the flat central region to the upper surfaces of the intermediate region, defining the contours of the radial structures. Within the radial structures, bacteria showed significant heterogeneity. Curli expression primarily occurred in cells located at the air interface, while its expression within the depth of the structures appeared limited. The outer region of the biofilm showed again a different topography, consisting of a thick mat of GFP expressing bacteria with no sign of the red curli signal (Fig. 6D).

The region-specific curli expression locations intrigued us, prompting further analysis of the biofilm's 3D structure. We analyzed a 3D reconstruction and axial-cross section from an area located at the interface of the central and intermediate regions (Fig. 6E). In the central region, curli signal was observed beneath the GFP expressing bacteria. As the radial structures emerged in the intermediate region, the location of the curli signal shifted to the upper surface. Underneath the curli-rich contours of the radial structures, GFP but not curli expressing bacteria were again seen (Fig. 6E). The localization of the curli signal in the upper part of the biofilm matrix was further confirmed by visualizing only the red channel of a cross-section of the intermediate region (Fig. 6F). The red curli signal clearly delineated the surface of the radial structures, showing significant vertical height variations. The curli signal appeared strongest in the walls of the radial structures with less defined signal on the top of the structures.

Taken together, by combining two non-interventional techniques, *i.e.* 2-photon imaging of ‘ready-labeled’ biofilms from the Ebba680-biofilm assay, we have provided novel, high-resolution information of the native 3D structure of UPEC12 biofilms. This revealed an extensive structural heterogeneity, particularly in curli location, in both the horizontal and vertical planes of the biofilm.

### 3.7 Curli architecture based on cellulose scaffolds causes increased surface hydrophobicity

**Fig. 7:**
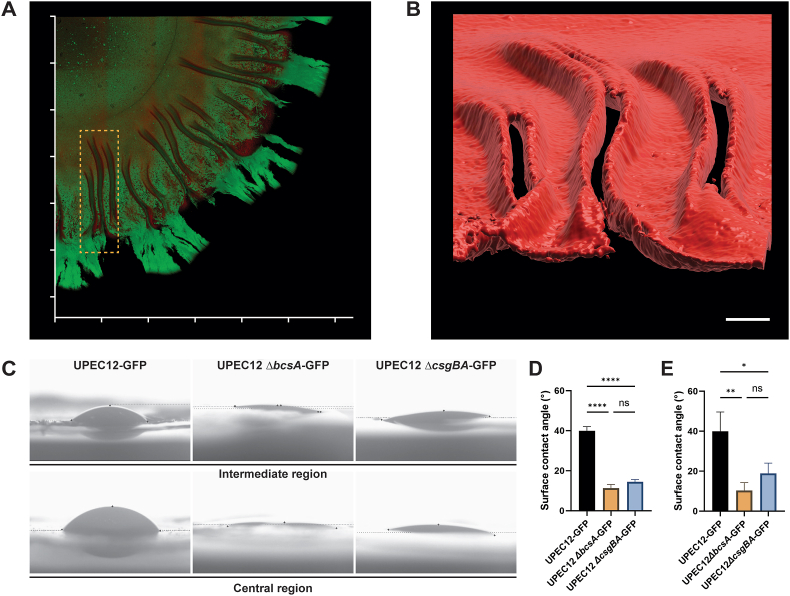
Curli expression and architecture causes increased surface hydrophobicity (A, B) Surface rendering of 3D reconstructions from 2-photon microscopy shows the curli architecture in the intermediate region of UPEC12-GFP biofilm grown for 72 h at 37 °C. (A) Duplicate of Fig. 6A, showing region (dotted box) with multiple radial structures selected for IMARIS surface rendering. Grid interval = 1 mm (B) Surface rendered image of the Ebba680-labeled curli signal (red) of the boxed region in (A), shown as an axially tilted image. Scale bar = 0.1 mm, representative image of n = 3. (C–E) Region-specific wettability of biofilms formed by UPEC12-GFP, UPEC12 Δ*bcsA*-GFP, and UPEC12 Δ*csgBA* -GFP grown for 72 h at 37 °C. (C) Images of water droplets positioned on the surfaces of the intermediate (top) and central (bottom) region of biofilms formed by indicated strains. Representative image of n = 3. (D–E) Quantitative analysis of surface contact angles of droplets in the (D) intermediate and (E) central regions of biofilms formed by indicated strains. n = 3. Statistical significance determined by one-way ANOVA. Statistical significance denoted by ns (not significant), *p ≤ 0.05, **p ≤ 0.01, ***p ≤ 0.001 or ****p ≤ 0.0001. (For interpretation of the references to colour in this figure legend, the reader is referred to the Web version of this article.)

To further analyze the curli heterogeneity within the radial structures of the intermediate region, we focused on an area containing three entire radial structures (Fig. 7A). To visualize the curli surface topography, we separated the red fluorescence channel and performed surface rendering in Imaris. An axially tilt of the surface rendering allowed radial structures to be viewed along their axis from the outer region towards the central region (Fig. 7B). The curli-rich radial structures showed dramatic vertical height above the agar and appeared to form crevasses-like structures in the biofilm. The heterogeneric curli expression was again observed. Close to the central region, the curli structures appeared to have an intact ‘roof’ which connected the vertical ridges. As the structures developed towards the outer/intermediate regions, the crevasses opened further and lacked this roof structure. At the border of the intermediate and outer region, a sharp delineation in the red curli signal representing the outer limit of curli expression in the biofilm was seen.

Surface rendering offered a unique opportunity to study the top layer of the unfixed biofilm, which in the intermediate region showed extensive presence of curli at the air interface. We hypothesized that this curli-rich layer may provide hydrophobic protection to cells in the biofilm. To evaluate this, we performed wettability testing, a standard method in material sciences when analyzing the hydrophobicity/hydrophilicity of surfaces (Supplementary Fig. 6). Using a goniometer, 1–2 μl water droplets were deposited onto the surface of defined regions of biofilms formed at 37 °C for 72 h (Fig. 6C). Based on the droplet features, the contact angles of each drop were measured. In the intermediate region of the wt biofilm (UPEC12-GFP), the mean contact angle was 40° (Fig. 6D). In contrast, the contact angle in the cellulose mutant UPEC12 *ΔbcsA*-GFP was 11.3°, suggesting a much higher degree of hydrophilicity in this biofilm. This was confirmed by visual observation of the deposited drop, which became partially absorbed into the biofilm. A similar pattern was also observed for the curli mutant UPEC12 *ΔcsgBA*-GFP, showing a contact angle of 14.5°. Thus, higher hydrophobicity was observed in biofilms formed by curli and cellulose expressing bacteria, compared to the mutants that lacked either curli or cellulose expression. This suggests a critical role of cellulose as an architectural scaffold of the biofilm, helping to orient the curli-rich layer in 3D, such that higher hydrophobicity of the biofilm is obtained.

We next analyzed the wettability of the flat, central region of the biofilms. In UPEC12-GFP biofilms, the central region showed contact angle of 39.9° (Fig. 6C and D). The cellulose mutant showed a super-hydrophilic central region with a contact angle of 10.3°, whereas the curli mutant showed 18.8°. The wettability pattern of the flat central region of biofilms formed by the three strains thus mimicked the patterns observed in the highly structured intermediate regions of corresponding strains. This infers that the radial macrostructures of the intermediate region contribute minimally, if at all, to the biofilm surface wettability. Instead, a comparatively hydrophobic surface of UPEC biofilms is achieved by coordinated expression of ECM components curli and cellulose, wherein cellulose provides an essential nanoscale scaffolding for the curli-rich layer. Collectively, while suggesting a need for cellulose-assisted curli orientation at the nanoscale to generate a comparatively hydrophobic ECM surface of UPEC biofilms, a possible role, if any, for the radial macrostructures in the intermediate region remains to be identified.

## 4 Discussion

Here, we used non-interventional approaches to study the dynamic, structural development of native biofilms by the clinical strain UPEC12 at physiological temperature of 37 °C. Taking advantage of the curli reporting qualities of Ebba680, real-time kinetic imaging, and high-resolution 2-photon imaging, we reveal a highly structured developmental process with heterogenous expression of curli in the horizontal and vertical planes of the biofilm. Region-specific radial structures, formed within a narrow 14–24 h time span, required coordinated expression of curli and cellulose. We showed these macrostructures to be insignificant for biofilm surface wettability. Instead, increased hydrophobicity of the biofilm, providing a hydro-protective shield to the bacteria, depended on cellulose-guided curli architecture at the nanoscale.

UPECs are considered good biofilm formers, yet we found great variation amongst strains, with only UPEC12 showing strong biofilm at 28 °C and 37 °C. Molecular analysis and spectrophotometric quantification of curli *in situ* suggest that the variation is primarily due to differences in curli production. However, synergy between curli and cellulose is needed in order to form the eye-catching phenotype indicative of the biofilm. All agar-based biofilm assays are, however, only model systems, unable to replicate the complex, hydrodynamically challenging *in vivo* environment of the urinary tract. In previous real-time *in vivo* studies, we identified a role for the Type 1 fimbriae in mediating inter-bacterial binding in bacterial macrocolonies, enabling bacterial colonization of kidney tubules in the face of shear stress [20]. To further understand the role of Type 1 fimbriae in bacterial colonization of host cells under flow, we used time-lapse microscopy in a proximal tubule-on-chip [31]. We found rapid macrocolony formation, dependent on Type 1 fimbriae to maintain their resistance to flow but interestingly, bacteria did not express curli in the first hours. Curli was only seen in the mature biofilm. An early-stage Type 1 dependence followed by later phase curli illustrate that biofilm formation, initiated from single bacterium adhesion, occurs via dynamic developmental processes requiring the involvement of different components throughout the maturation process. This supports previous work in the field, showing that many biofilm related processes are related to stationary phase physiology of the bacteria [28]. Our studies were performed using CFT073 *rpoS*^*-*^, found to harbor an amber mutation in *rpoS,* as well as the wt CFT073 [20,31]. Interesting, despite evidence of biofilm-like behaviors in *in vivo* and proximal tubule-on-chip models, neither strain gave a biofilm phenotype in the CR- or Ebba680-biofilm assays. This highlights that the biofilm-forming capacity of strains heavily depends on which microenvironment they are in, and that care should be taken when judging a strain to be a biofilm producer or not.

Real-time visualization of UPEC12 biofilm formation showed active expansion of the biofilm in the horizontal plane within the first 24 h, forming a flat central and a structured intermediate region. Radial structure formation synchronized with curli and cellulose expression. Curli expression particularly was orders of magnitude higher in the intermediate region as compared to the central region, pointing to region-specific expression patterns within the biofilm. The ability to watch the biofilm development in real-time led also to other unexpected observations. One was that the central region reduced in size upon development of the intermediate radial structures, becoming smaller than the initial inoculum. We also noted a distinct red signal emanating from the expanding intermediate region of the biofilm prior to the appearance of the radial structures. This implies that curli expression may precede that of cellulose and that only once both structural components are present, the radial structures form. Our data supports previous work, showing that precis spatial assembly of curli and cellulose is critical to the morphology of the macroscopic 3D patterns termed wrinkled, rugose, or rdar [7,28,32]. In addition, our data adds new dynamic insights to the development of the rdar phenotype, which appears fully formed already after 24 h. After this timepoint, the outer region emerged and non-ECM expressing bacteria continued to slowly expand horizontally. Our data highlight that the bacterial cells of a biofilm community are incredible heterogenic, showing spatial- and temporal-specific genetic expression patterns. The ability to combine dynamic, kinetic data with direct read-outs of ECM expression opens the door for a new generation of understanding of this bacterial lifestyle.

The spatial-temporal multi-cellular behavior of biofilm bacteria is reminiscent of tissues in eukaryotic organisms. Considering the biofilm as a bacterial ‘tissue’, we employ 2-photon intravital imaging, which we previously used to image UPEC infection within a living kidney [20,33], to image deep into the native biofilm structure without the need for fixation, cryopreservation, slicing or other interventions. The resulting data gives a high-resolution picture of the native biofilm structure, with Ebba680 as a reporter for curli. Intriguingly, curli location varied in different regions of the biofilm. In the central region, the curli signal was located below a layer of green bacterial cells, while it shifted in the intermediate region to the apical aspect of the biofilm and to curli rich ridges. Importantly, the high-resolution images show that the radial structures are distinct folds with curli only located on the upper surfaces and walls. This supports previous work where agar biofilm has been shown to buckle or fold into the distinct rdar phenotypes based on complementary actions of curli and cellulose expression in the macrocolonies [6,7]. Our microscopy data also supports that the base layers of the intermediate region appear to express less ECM [34,35]. Areas underneath the curli rich structures are filled with GFP bacteria lacking curli expression, demonstrating vertical heterogeneity of the bacterial population. This intricate pattern mimics findings from our previous work *in vivo,* showing the importance of differential expression of adhesion organelles in the biofilm-like bacterial community colonizing the peripheral vs central areas of a single renal tubule [20]. Collectively, it highlights the exquisite control of differential gene expression by bacteria within the complex superstructures of biofilm. Further investigation into this biofilm heterogeneity is now underway by non-interventional, combined high-resolution and time-lapse imaging.

Radial structure formation has long been used as a read-out for biofilm formation, yet no one knows if these macroscale structures confer biological functions to the biofilm community. We hypothesized a role of the radial structures in surface wettability, *i.e.* the establishment of hydrophobic outer surfaces, such that the biofilm resists wetting by aqueous liquids. Our water contact angle measurements rather showed that the nano-structural features defined by cellulose-assisted structuring of curli, are required for establishment of the relative surface hydrophobicity of UPEC12 biofilms. The relative water excluding nature is thus an emergent property of the constituents as well as the nano-architecture of the ECM, synergistically formed by cells, curli fibrils, and cellulose. Data from this first wettability measurement on native, intact UPEC biofilms presents a new paradigm in contrast to data obtained from extracted bacterial cells and curli fibers from disrupted biofilms [36,37]. If UPEC biofilms in the urinary tract exhibit a relatively hydrophobic surface, it may have multiple consequences from a medical perspective. Wetting resistance may hinder the action of host-related antimicrobial compounds (e.g. LL-37^22^) and antibiotics by limiting the access to the biofilm interior. Also, by relative exclusion of the biofilm from aqueous environment, resistance to liquid wetting may assist in preventing dislodging of biofilms due to urine flow. The view of ECM as a simple physical barrier acting through size exclusion thus ought to be modified, as our data propose an important role of surface properties, such as charge and nano-structural arrangements, for the wettability, and ultimately the biofilms’ accessibility of water-soluble antimicrobial compounds.

While the Ebba680-biofilm assay has delivered extensive new insights on *Salmonella* and now UPEC biofilms, an emerging technique such as this requires pre-validation of the binding target. Depending on bacteria of interest, this would require use of defined mutants or purified components, which may limit the applicability of the method. However, multiple variants of the EbbaBiolight-series of optotracers are commercially available. This broadens the range of potential targets, thereby opening for exciting possibilities to extend the spatial-temporal understanding of biofilms formed by many bacteria and fungi.

## 5 Conclusions

Non-destructive methods enabling longitudinal kinetics and 3D structural analysis provides new insights in biofilm formation. Tissue-like UPEC biofilms show genetic, phenotypic, and morphologic heterogeneities. Biofilms form via a maturation process wherein bacterial cells build sub-environments within the community. The *in vitro* biofilm-forming capacity of UPEC strains varies widely, highlighting that model systems do not reflect the *in vivo* situation. Whether radial structures are significant for UPEC biofilm formation in any environment or represent artefacts of the model system is unclear. The nano-structural arrangements of ECM components and the surface charge determine, however, the biofilms’ wettability. We envision that deeper understanding of how to tune the wetting of a biofilm will enable future development of antimicrobial therapies targeting the sessile biofilm lifestyle.

## Disclosures

TZ, SR, and KM declare no competing interests. A.R.D. is co-inventor of patents relevant to this work. Intellectual properties are owned by Richter Life Science Development AB, founded by A.R.D., who also has an engagement in Ebba Biotech AB, which commercializes optotracers for uses as described in this article.

## Funding

All authors acknowledge support from AIMES – Center for the Advancement of Integrated Medical and Engineering Sciences (www.aimes.se) at 10.13039/501100004047Karolinska Institutet (1–249/2019) and 10.13039/501100004270KTH Royal Institute of Technology (VF-2019–0110), 10.13039/100030785Getinge AB (4–1599/2018). The work was further supported by the 10.13039/501100004359Swedish Research Council (2019-01460 and 2023–02078, ARD) and (2020-01790, KM) and 10.13039/501100004047Karolinska Institutet KID funding (2019-00917). Funding sources had no direct involvement in any aspects of the work.

## CRediT authorship contribution statement

**Tianqi Zhang:** Writing – review & editing, Writing – original draft, Methodology, Investigation, Formal analysis, Data curation, Conceptualization. **Sanhita Ray:** Writing – review & editing, Methodology, Investigation, Formal analysis, Data curation. **Keira Melican:** Writing – review & editing, Writing – original draft, Supervision, Resources, Project administration, Methodology, Funding acquisition, Formal analysis, Conceptualization. **Agneta Richter-Dahlfors:** Writing – review & editing, Writing – original draft, Supervision, Resources, Project administration, Methodology, Funding acquisition, Formal analysis, Conceptualization.

## Declaration of competing interest

The authors declare the following financial interests/personal relationships which may be considered as potential competing interests:

Agneta Richter-Dahlfors reports a relationship with Ebba Biotech AB that includes: board membership and equity or stocks. Agneta Richter-Dahlfors has patent licensed to Ebba Biotech AB. Intellectual properties relevant to this work is licensed by Ebba Biotech AB from the Richter Life Science Development AB, founded and co-owned by A. Richter-Dahlfors. If there are other authors, they declare that they have no known competing financial interests or personal relationships that could have appeared to influence the work reported in this paper.

## Data Availability

No data was used for the research described in the article.
