# Supplementary material for: The maturation of native uropathogenic Escherichia coli biofilms seen through a non-interventional lens
Source: Biofilm. 2024 Jul 6;8:100212. doi: 10.1016/j.bioflm.2024.100212 (PMC11305213; doi:10.1016/j.bioflm.2024.100212)
Supplement: Multimedia component 1 [file mmc1.docx]

**SUPPLEMENTARY INFORMATION**

**The maturation of native uropathogenic *Escherichia coli* biofilms seen through a non-interventional lens**

Tianqi Zhang^a,b^, Sanhita Ray^a,b^, Keira Melican^a,b,^*, Agneta Richter-Dahlfors^a,b,^*

^a^ AIMES - Center for the Advancement of Integrated Medical and Engineering Sciences at Karolinska Institutet and KTH Royal Institute of Technology, Stockholm, Sweden

^b^ Department of Neuroscience, Karolinska Institutet, Stockholm, Sweden

* These authors contributed equally

**Page index Page**

Supplementary Table 1 2

Supplementary Fig. 1 3

Supplementary Fig. 2 4

Supplementary Fig. 3 5

Supplementary Fig. 4 6

Supplementary Fig. 5 7

Supplementary Fig. 6 8

Supplementary Movies 1-3, captions 9

**Supplementary Table 1. Strains and plasmids**

| **Strains** | **Characteristics** | **Source** |
| --- | --- | --- |
| W3110 | laboratory *Escherichia coli* W3110 | *E. coli* Genetic Resources at Yale CGSC, The Coli Genetic Stock Center |
| CFT073 *rpoS^-^* | Uropathogenic *Escherichia coli* CFT073 with mutation in *rpoS* | Hryckowian et al., 2015 |
| CFT073 | Uropathogenic *Escherichia coli* CFT073 | Mobley et al., 1990 |
| UPEC12 (UPEC strain No. 12) | Uropathogenic *Escherichia coli* isolate from a child with pyelonephritis | Kai-Larsen et al., 2010 |
| UPEC12 *ΔbcsA* | UPEC12 *bcsA*::Km^R^ | Kai-Larsen et al., 2010 |
| UPEC12 *ΔcsgBA* | UPEC12 *csgBA*::Km^R^ | Kai-Larsen et al., 2010 |
| UPEC12-GFP | UPEC12 carrying vector for green fluorescence protein expression pFPV25.1 | This work |
| UPEC12 *ΔbcsA*-GFP | UPEC12 *ΔbcsA* carrying vector for green fluorescence protein expression pFPV25.1 | This work |
| UPEC12 *ΔcsgBA*-GFP | UPEC12 *ΔcsgBA* carrying vector for green fluorescence protein expression pFPV25.1 | This work |
|  |  |  |
| **Plasmids** | **Characteristics** | **Source** |
| pFPV25.1* | Vector for green fluorescent protein expression and ampicillin resistance (Amp^R^) | Valdivia and Falkow, 1996 |
|  |  |  |
| **Primers** | **Sequence (5'-3')** |  |
| *rpoD* forward | ATGGTTGAAGCGAACTTGCG |  |
| *rpoD* reverse | TCAACCGCTTTCATCAGACC |  |
| *csgD* forward | ACCGCGACATTGAAAACTGG |  |
| *csgD* reverse | TGCAACCCATTGACAACACG |  |
| *csgA* forward | TGATCTGACCATTACCCAGCAC |  |
| *csgA* reverse | ATCAAGAGTAGCGCTGTTGC |  |
| *bcsA* forward | TTGTGCAGTGTACGTTTGCC |  |
| *bcsA* reverse | ACCGAAGGCGCAAATTC |  |

*pFPV25.1 was a gift from Raphael Valdivia (Addgene plasmid #20668; <http://n2t.net/addgene:20668>; RRID: Addgene_20668)

**References**

Hryckowian, A.J., Baisa, G.A., Schwartz, K.J., Welch, R.A., 2015. dsdA Does Not Affect Colonization of the Murine Urinary Tract by Escherichia coli CFT073. PloS One 10, e0138121. https://doi.org/10.1371/journal.pone.0138121

Kai-Larsen, Y., Lüthje, P., Chromek, M., Peters, V., Wang, X., Holm, A., Kádas, L., Hedlund, K.-O., Johansson, J., Chapman, M.R., Jacobson, S.H., Römling, U., Agerberth, B., Brauner, A., 2010. Uropathogenic Escherichia coli modulates immune responses and its curli fimbriae interact with the antimicrobial peptide LL-37. PLoS Pathog. 6, e1001010. https://doi.org/10.1371/journal.ppat.1001010

Mobley, H.L., Green, D.M., Trifillis, A.L., Johnson, D.E., Chippendale, G.R., Lockatell, C.V., Jones, B.D., Warren, J.W., 1990. Pyelonephritogenic Escherichia coli and killing of cultured human renal proximal tubular epithelial cells: role of hemolysin in some strains. Infect. Immun. 58, 1281–1289. https://doi.org/10.1128/iai.58.5.1281-1289.1990

Valdivia, R.H., Falkow, S., 1996. Bacterial genetics by flow cytometry: rapid isolation of Salmonella typhimurium acid-inducible promoters by differential fluorescence induction. Mol. Microbiol. 22, 367–378. https://doi.org/10.1046/j.1365-2958.1996.00120.x

**Supplementary Figure 1**


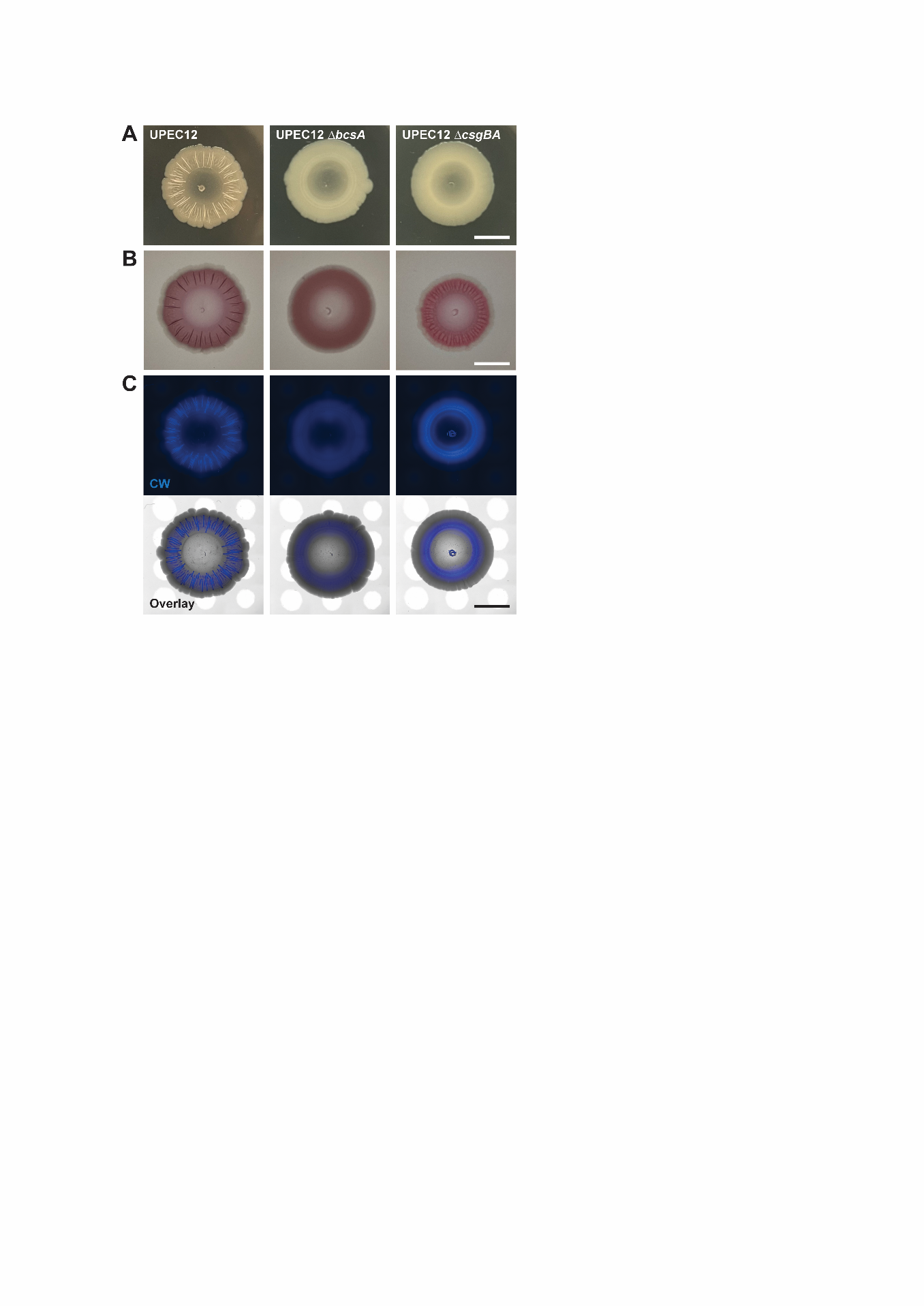


**Biofilms formed by UPEC12 and isogenic mutants on Congo red and Calcofluor white biofilm assays.** (A-B) Photographs of UPEC12, UPEC12 *ΔbcsA* (curli+, cellulose-) and UPEC12 *ΔcsgBA* (curli-, cellulose+) on (A) LB agar w/o salt and (B) CR-biofilm assay at 37 °C for 72 h. Scale bar = 5 mm. Representative images of n = 3. (C) Wide-field fluorescence imaging of UPEC12, UPEC12 *ΔbcsA* (curli+, cellulose-) and UPEC12 *ΔcsgBA* (curli-, cellulose+) on Calcofluor white (CW)-biofilm assay at 37 °C for 72 h. LB agar w/o salt was supplemented with 2 μl CW stain (Calcofluor White M2R (1 g/l), Evans blue (0.5 g/l), Sigma-Aldrich, Stockholm, Sweden) per ml agar. The CW image, representing CW-labeled cellulose (blue) imaged in the DAPI channel, is shown separately (upper row) and in overlay with the brightfield image (lower row). Scale bar = 5 mm. Representative images of n = 3.

**Supplementary Figure 2**


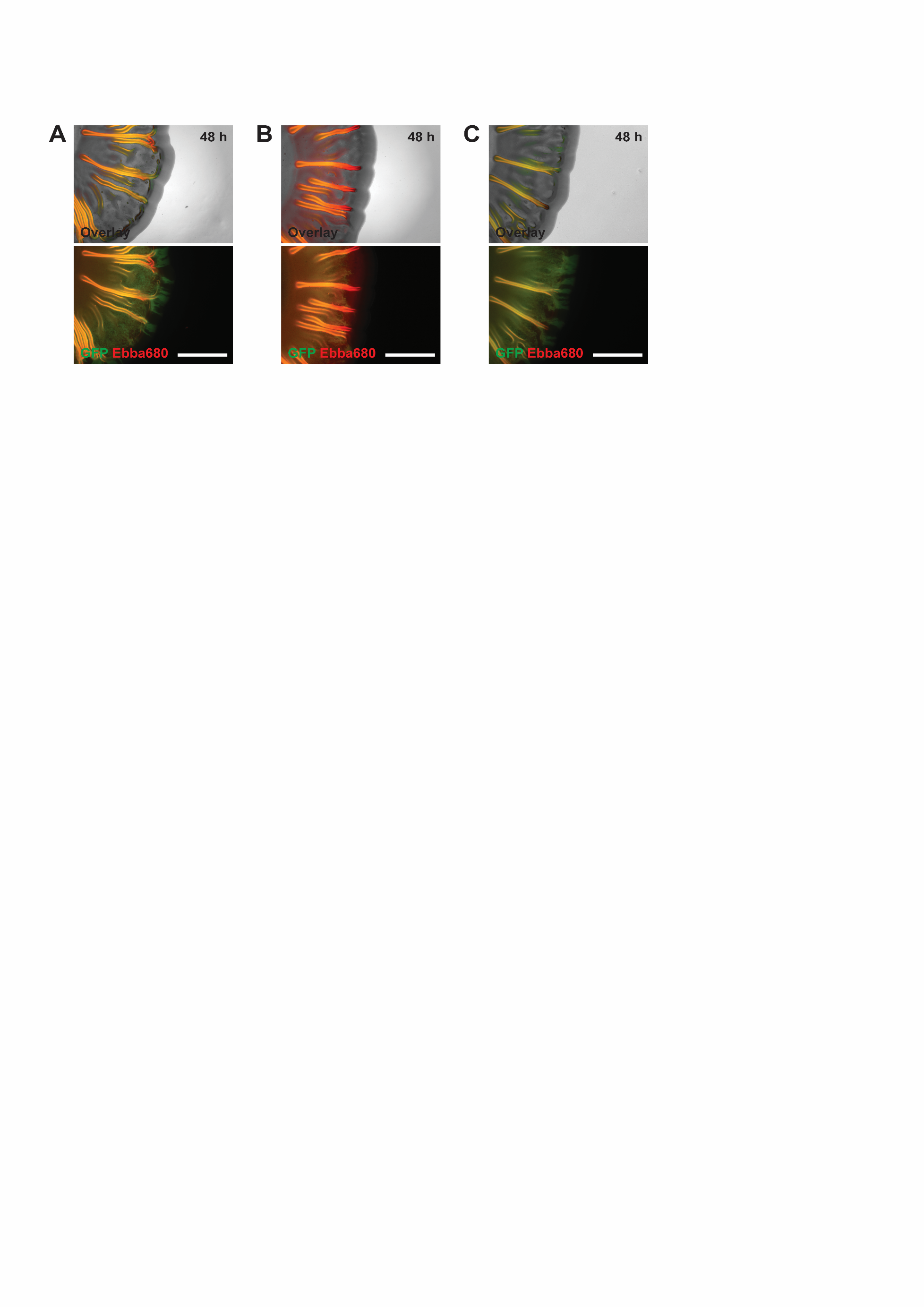


**UPEC12-GFP biofilm at 48 h.** Wide-field fluorescence imaging of UPEC12-GFP biofilm on Ebba680-biofilm assay grown for 48 h at 37 °C. The spatial distribution of UPEC12-GFP (green, GFP channel) and Ebba680-labeled curli (red, propidium iodide channel) shown separately and in overlays with respective brightfield image. (A-C) represents the 48 h time point from 3 experiments. (A) correlates to Fig. 4 and Supplementary Movie 1, (B-C) originate from Supplementary Movies 2-3, respectively. Scale bar = 2 mm.

**Supplementary Figure 3**


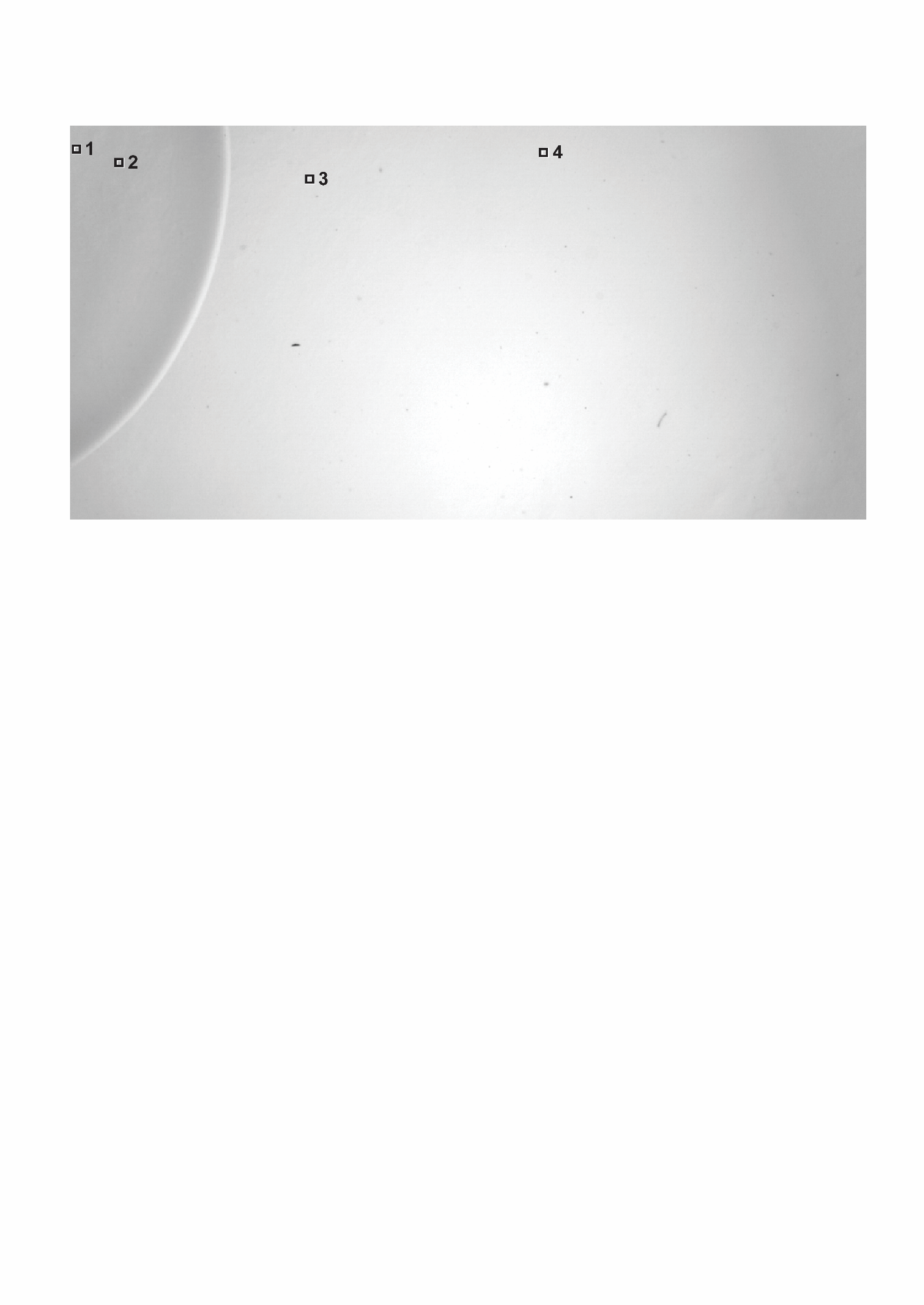


**Locations of ROI 1-4 at time 0 h.** Brightfield image showing position of ROIs, selected from the 72 h endpoint shown in Figure 4 A, on and outside the bacterial inoculum drop at the 0 h timepoint. RFU values within ROI 1-4 tracked through the 72 h growth period are shown in Figure 4 C-F, respectively.

**Supplementary Figure 4**


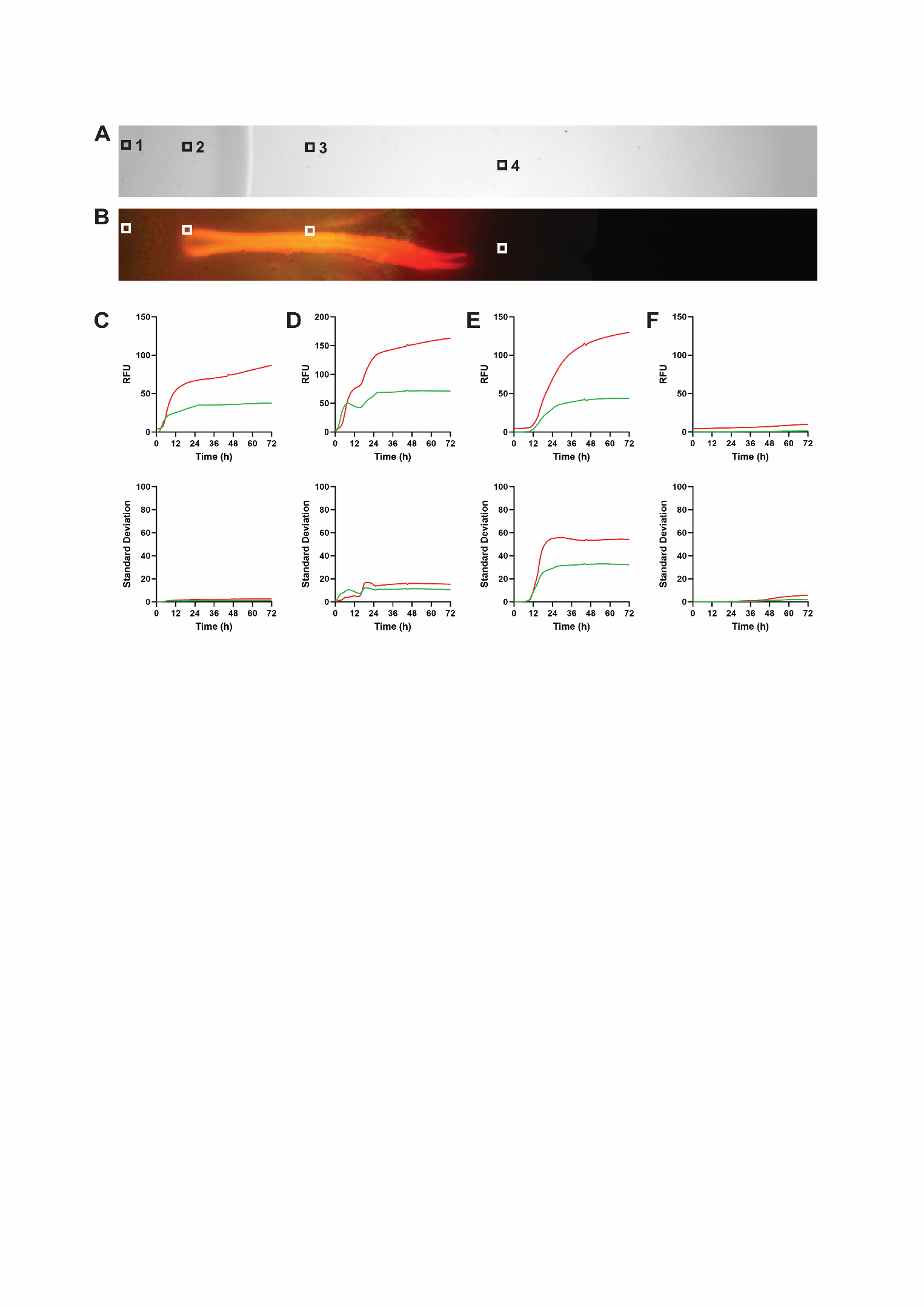


**Replicate dataset #2 for kinetics shown in Figure 4.** Image data originate Supplementary Movie 2. (A) Brightfield image showing position of chosen ROI 1-4 at 0 h timepoint. RFU values within these ROIs are tracked through the 72 h period over which the biofilm grows outwards from this drop. (B) Fluorescence image at 72 h showing position of chosen ROIs on fully formed biofilms. (C-F) Kinetics dataset for ROI 1-4 as described for Figure 4 C-F.

**Supplementary Figure 5**


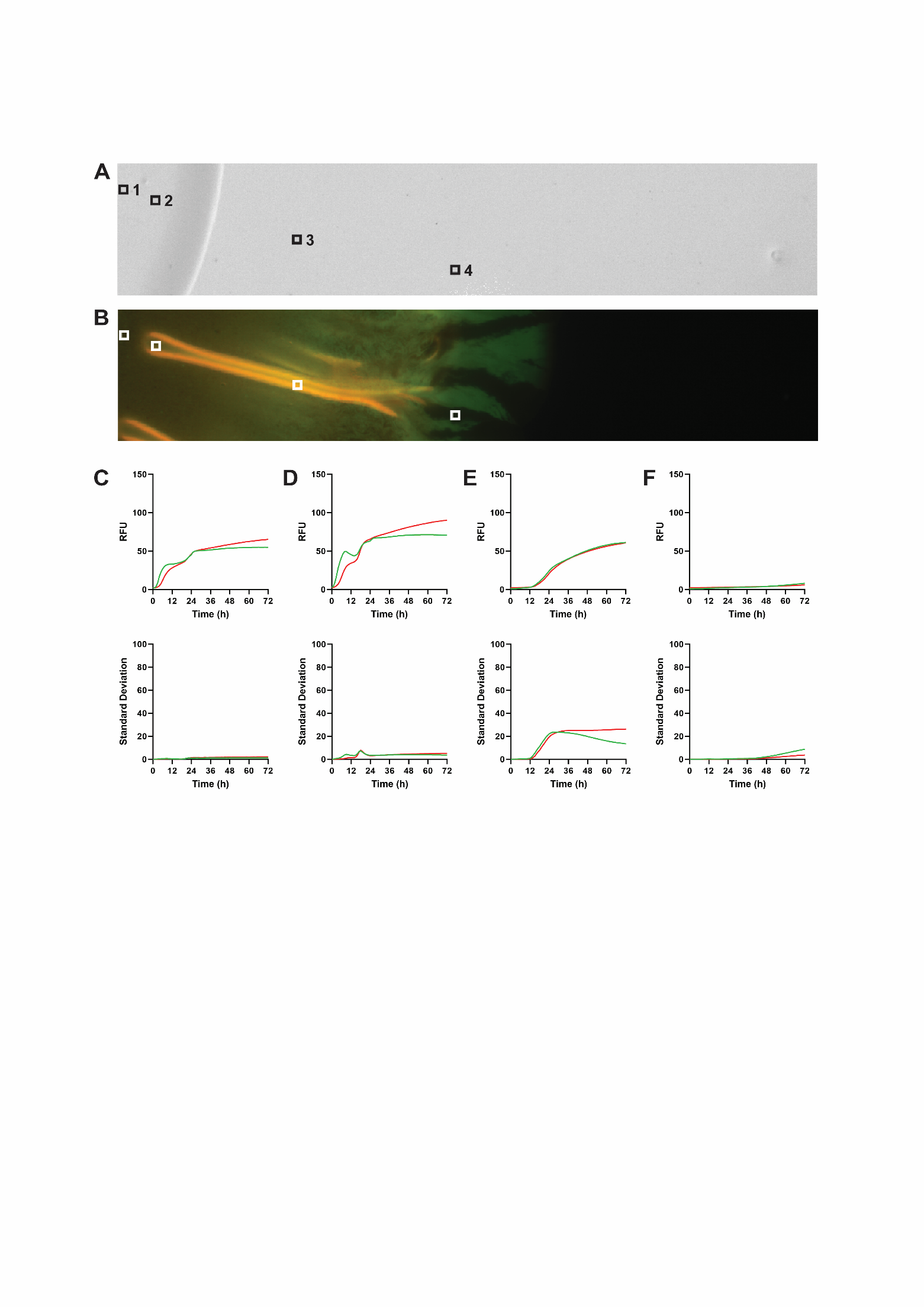


**Replicate dataset #3 for kinetics shown in Figure 4.** Image data originate Supplementary Movie 3. (A) Brightfield image showing position of chosen ROI 1-4 at 0 h timepoint. RFU values within these ROIs are tracked through the 72 h period over which the biofilm grows outwards from this drop. (B) Fluorescence image at 72 h showing position of chosen ROIs on fully formed biofilms. (C-F) Kinetics dataset for ROI 1-4 as described for Figure 4 C-F.

**Supplementary Figure 6**


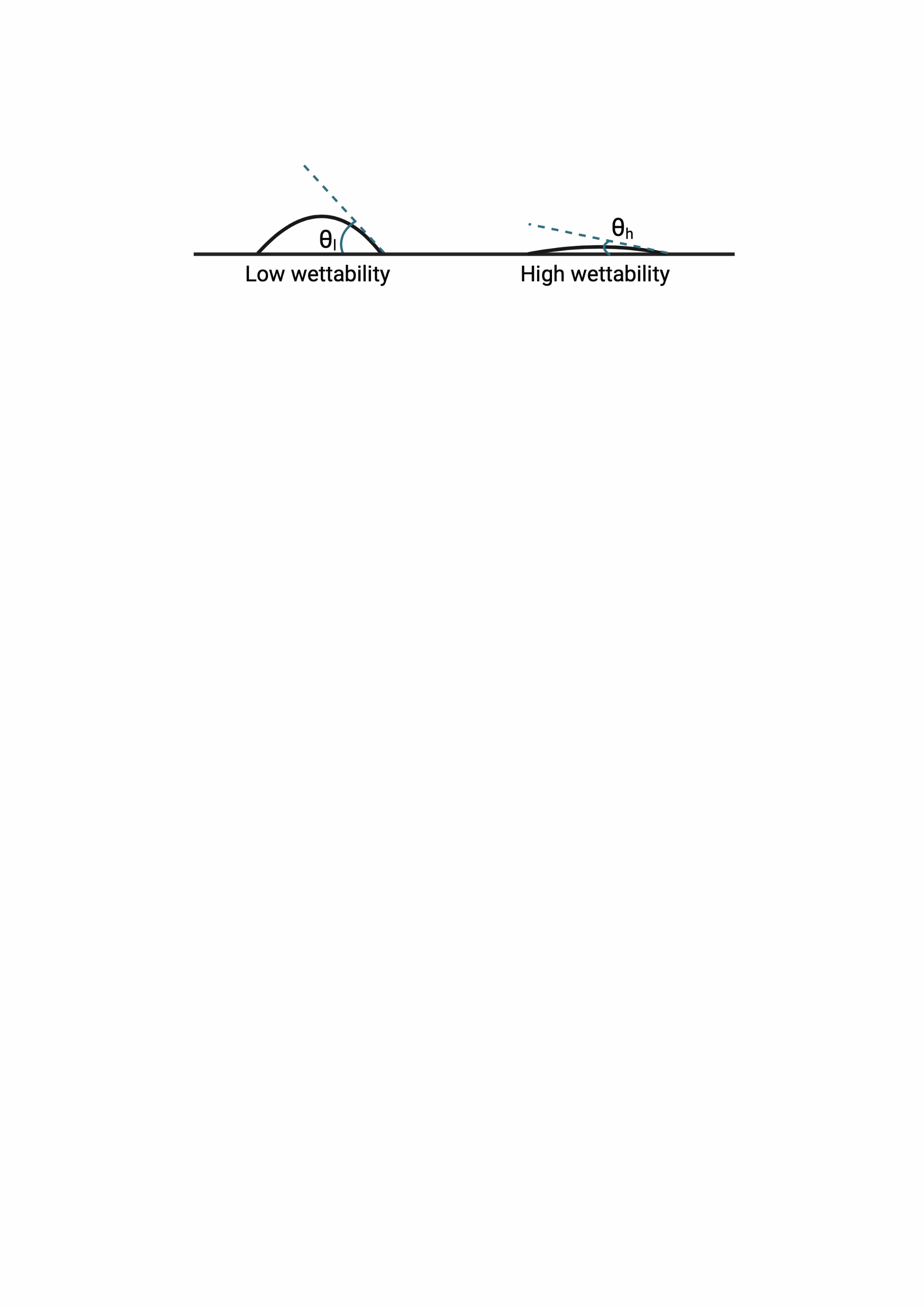


**Schematic showing the basics of wettability measurement.** Example droplet profiles on surfaces with low and high wettability. High contact angle (θ_l_) correlates to low wettability of the surface. Low contact angle (θ_h_) correlates to high wettability of the surface. When the contact angle is near or less than 10° the surface is considered super-hydrophilic.

**Supplementary Movie captions**

**Supplementary Movie 1. The kinetic development of UPEC12-GFP biofilm, repeat #1.** Time-lapse wide-field fluorescence imaging of UPEC12-GFP biofilm on Ebba680-biofilm assay grown for 72 h at 37 °C. The spatial distribution of UPEC12-GFP (green, GFP channel) and Ebba680-labeled curli (red, propidium iodide channel) shown separately and in overlays with respective brightfield image. Scale bar = 2 mm. Relates to Figure 4.

**Supplementary Movie 2. The kinetic development of UPEC12-GFP biofilm, repeat #2.** Time-lapse wide-field fluorescence imaging of UPEC12-GFP biofilm on Ebba680-biofilm assay grown for 72 h at 37 °C. The spatial distribution of UPEC12-GFP (green, GFP channel) and Ebba680-labeled curli (red, propidium iodide channel) shown separately and in overlays with respective brightfield image. Scale bar = 2 mm. Relates to Figure 4 and Supplementary Figure 3.

**Supplementary Movie 3. The kinetic development of UPEC12-GFP biofilm, repeat #3.** Time-lapse wide-field fluorescence imaging of UPEC12-GFP biofilm on Ebba680-biofilm assay grown for 72 h at 37 °C. The spatial distribution of UPEC12-GFP (green, GFP channel) and Ebba680-labeled curli (red, propidium iodide channel) shown separately and in overlays with respective brightfield image. Scale bar = 2 mm. Relates to Figure 4 and Supplementary Figure 4.
